# Modelling southern elephant seals *Mirounga leonina* using an individual-based model coupled with a dynamic energy budget

**DOI:** 10.1371/journal.pone.0194950

**Published:** 2018-03-29

**Authors:** Merel Goedegebuure, Jessica Melbourne-Thomas, Stuart P. Corney, Clive R. McMahon, Mark A. Hindell

**Affiliations:** 1 Institute for Marine and Antarctic Studies, University of Tasmania, Private Bag 129, Hobart, Tasmania 7000 Australia; 2 Antarctic Climate and Ecosystems Cooperative Research Centre, University of Tasmania, Private Bag 80, Hobart, Tasmania 7000 Australia; 3 Australian Antarctic Division, Channel Highway, Kingston Tasmania 7050 Australia; 4 Sydney Institute of Marine Science, 19 Chowder Bay Road, Mosman, New South Wales 2088, Australia; Institute of Zoology, CHINA

## Abstract

Higher trophic-level species are an integral component of any marine ecosystem. Despite their importance, methods for representing these species in end-to-end ecosystem models often have limited representation of life histories, energetics and behaviour. We built an individual-based model coupled with a dynamic energy budget for female southern elephant seals *Mirounga leonina* to demonstrate a method for detailed representation of marine mammals. We aimed to develop a model which could i) simulate energy use and life histories, as well as breeding traits of southern elephant seals in an emergent manner, ii) project a stable population over time, and iii) have realistic population dynamics and structure based on emergent life history features (such as age at first breeding, lifespan, fecundity and (yearling) survival). We evaluated the model’s ability to represent a stable population over long time periods (>10 generations), including the sensitivity of the emergent properties to variations in key parameters. Analyses indicated that the model is sensitive to changes in resource availability and energy requirements for the transition from pup to juvenile, and juvenile to adult stage. This was particularly the case for breeding success and yearling survival. This model is suitable for use as a standalone tool for investigating the impacts of changes to behaviour and population responses of southern elephant seals.

## Introduction

Models are important tools for understanding and predicting changes in ecosystem state, and informing management (*e.g*. [1, 2, 3]). However, the optimal level of detail with which to model specific ecosystem components depends on the aim of the model; detailed representations of ecosystem components can increase the cost associated with development and use of models, and intermediate levels of complexity can improve the predictive capacity of models [4]. Deciding on the necessary level of complexity required in a model is important; recent work has shown that the level of detail used for representations of higher trophic-level species such as seabirds and marine mammals can alter ecosystem-level responses to change, and can influence model predictions for single- [5, 6] and multi-species models [7]. In this regard, to achieve effective ecosystem based management, models should ideally be developed in such a way that the representation of individuals can be used for population and ecosystems ecology [8]. Moreover, when developing a model to examine likely outcomes of future scenarios, or for conservation and management purposes, adding detail on the target species is an important consideration, particularly as behaviour (both that of the individual, as well as that of the population) and energy intake and expenditure are factors that are known to be influenced by environmental changes (see [9]). Consequently, an essential component to ecosystem based management is the ability to quantify prey consumption by predators as this information can be used in the development of broader scale ecosystem models and management approaches (see [2, 9, 10]).

There have been a range of single-species models with bioenergetics components developed for marine predators. Langton *et al*. [11] developed an individual-based model (IBMs: [12, 13]) for the common guillemot *Uria aalge*. This model includes fine scale energetic representations for adults and their chick during the breeding season to address theoretical ecological questions and inform marine spatial management. Pavlova *et al*. [14] designed an IBM to estimate food consumption by polar bears *Ursus maritimus* using known blubber content of East Greenland seals (the main prey species), and provide insight into polar bear energetics. Southwell *et al*. [9] developed a bioenergetics model for Adélie penguins *Pygoscelis adeliae* to predict prey consumption during their breeding season. Bejarno *et al*. [15] developed a conceptual bioenergetics model to estimate energy requirements of the bottlenose dolphin *Tursiops truncatus*, which includes estimation of prey biomass consumption using three different methods.

To improve the detailed representation of energetic use by species, and to explore population wide responses to perturbation, the use of DEB-IBMs (*sensu* [16]) has been suggested for representation of higher trophic-level predators with complex life histories [7, 17]. These models incorporate dynamic energy budget (DEB) theory [18, 19] within IBMs. Dynamic energy budget theory [18] uses a deterministic approach to model the use and flow of energy by individuals and incorporates an individual’s assimilation and energy use for growth, maintenance, and reproduction [18, 20, 21] throughout its life-cycle (see also review by [22]). Individual-based modelling enables the study of individual interactions, system behaviours and complex multi-level interactions within the system [12, 13, 23, 24].

**Fig 1:**
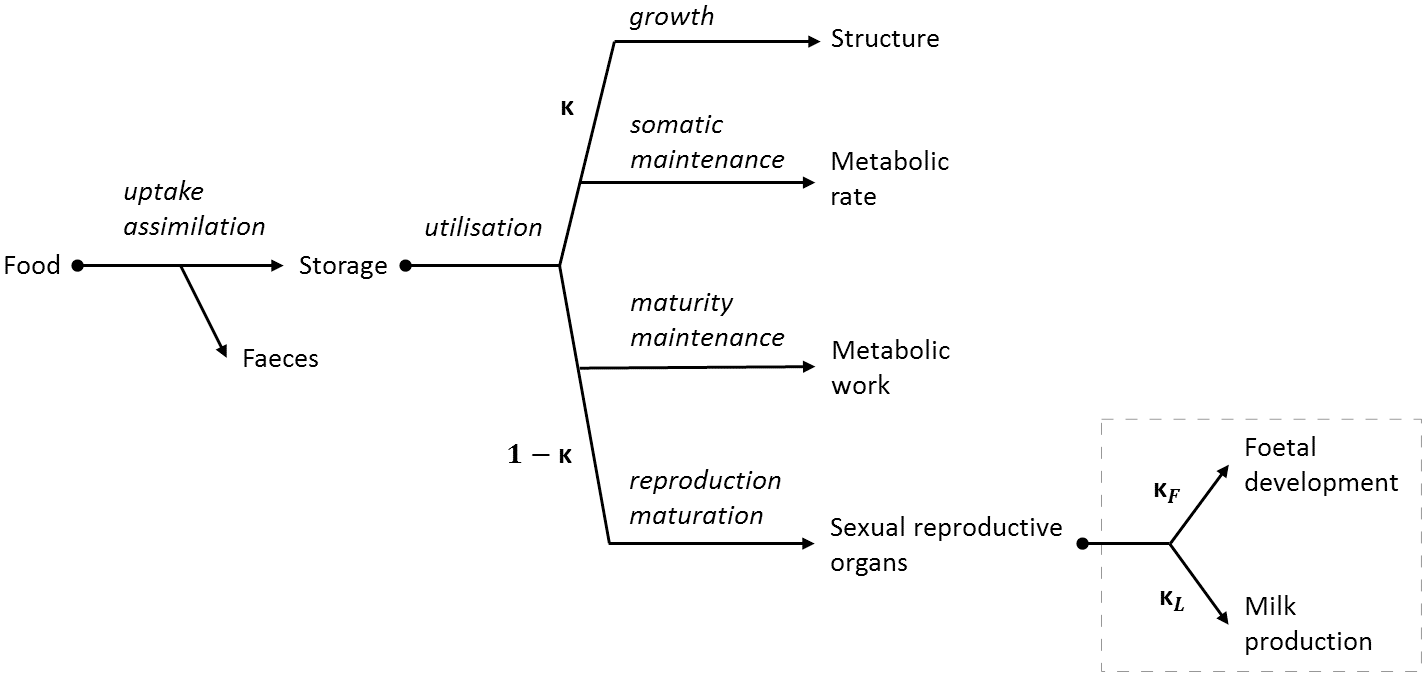
A dynamic energy budget model representation for a general organism. A dynamic energy budget model representation (modified from Goedegebuure *et al*. [7], following Kooijman [18] and Roberts [30]) for a general organism, with the additional resource allocation for foetal development, and lactation by mothers (dotted box). As food is ingested, energy is extracted and added to the reserves (storage). It is then utilised for growth, somatic maintenance, maturity maintenance, and reproduction maturation. The kappa-rule [18] gives absolute priority to energy allocation for growth and somatic maintenance. While the remaining energy (1 − *κ*) is utilised for maturation (embryos and juveniles), reproduction, and maturity maintenance (adults). Reproductive energy is allocated to foetal development *κ*_*F*_, or milk production *κ*_*L*_, depending on the pregnancy or lactation status of the individual.

The DEB-IBM framework combines the deterministic aspects of DEB theory (Fig 1) and the stochasticity of IBMs to study effects at a population level [16]. It is based on well-tested physiological principles to represent individuals throughout their life cycle, and has been applied to a number of species including water-fleas *Daphina magna* [16, 25, 26]; oysters *Crassostrea gigas* [27]; zebrafish *Danio rerio* [28]; Antarctic krill *Euphausia superba* [29], and anchovies *Engraulis encrasicolus* [17].

As yet, no DEB-IBM has been specifically developed for mammals, however, the framework is well suited to model these species considering its potential to include complex life histories and breeding behaviour, as well as its ability to analyse population characteristics and predator-prey interactions [17]. Thus, the goal of this paper is to demonstrate the use of DEB-IBMs for the detailed representation of a higher trophic-level predator. We present a DEB-IBM developed for female southern elephant seals *Mirounga leonina*; an abundant top predator of the Southern Ocean.

We included only female southern elephant seals in the model, as these make up the largest part of the population ([31] and S1 Table), and are a crucial component in the survival of the species considering they singularly nurse their pups (as opposed to sharing this responsibility with a partner, as is the case for penguins, *e.g*.; [32]). The male population does not strongly influence the overall population trajectory (males make up only 36% of the adult population (S1 Table), and only around 8% of males actually sire pups in a given year (which is based on the number of males over the age of 9, and the number of pups born in that year)). As such the population trajectory of southern elephant seals is only weakly dependent on the population size of males.

There are valid arguments as to why males should be included in population models for populations where male and female dynamics may differ (see [33, 34])—specifically for matrix population models and for understanding extinction risks—as opposed to assuming that populations can be represented based on females only [35]. However many of the arguments for including both sexes in population models assume that both sexes forage in similar environments, which is not the case for the majority of southern elephant seals (see [36, 37] for foraging and annual haul-out patterns). Additionally the assumption for having two-sex models for polygynous species, such as southern elephant seals has been shown to be important only when both male and female survival rates are low, as changes in male survival rates (when that of females stays high) has limited impact on population growth [34]; the survival rates of male southern elephant seals is significantly lower than that of females [31].

As this DEB-IBM focusses on the population change over time, not just on the energy flow, we choose not to explicitly model male seals. For simplicity in the model it is assumed that all pups are born female, and remain female. Although at birth the ratio of males to females is equal, overall the population is comprised of approximately 36% males and 64% females (see S1 Table, up to the age of 15 as this is the maximum observed age of male southern elephant seals). The energy that mothers expend on producing male pups at a ratio of 1:1 is accounted for in the model by increasing the breeding threshold (see section Thresholds for puberty, breeding and death in Model modifications), to ensure that we have (roughly) half the observed number of births.

Global numbers of southern elephant seals have increased in recent years following recovery from commercial exploitation, however this trend is not prominent in all sub-populations [38]. The population at Macquarie Island has been in decline since the 1960s [38] at a rate of -1.45% per annum [39]. We have used the data collected from longitudinal studies on southern elephant seals on Macquarie Island for the model development. The specific aims of the study were to develop a DEB-IBM that could i) accurately simulate energy use, life histories and breeding traits of female southern elephant seals in an emergent manner, while ii) projecting a stable population over time, and iii) be used to evaluate the sensitivity of the emergent demographic properties to variations in key parameters.

## Materials and methods

### Study species

Southern elephant seals forage throughout the Southern Ocean [38, 40] and are extreme capital breeders (they accumulate energy prior to breeding, and provision young by using only those stores [41, 42]). They have pronounced sexual dimorphism (females up to 800 kg and 2.8 m in length; males up to 3000 kg and 3.5 m [43, 44]). For females, breeding starts at the age of three [45], with optimal breeding after the age of four [46], while somatic growth continues to the age of six [45, 46]. Males reach sexual maturity at the age of five; however competition with more dominant bulls prevents these sub-adults from successfully mating. Somatic growth for males continues until seven years old, at which stage they may succeed in overpowering previously dominant bulls, creating a harem, and reproducing [44, 45]. The maximum recorded age of female southern elephant seals is 23 years [47].

**Fig 2:**
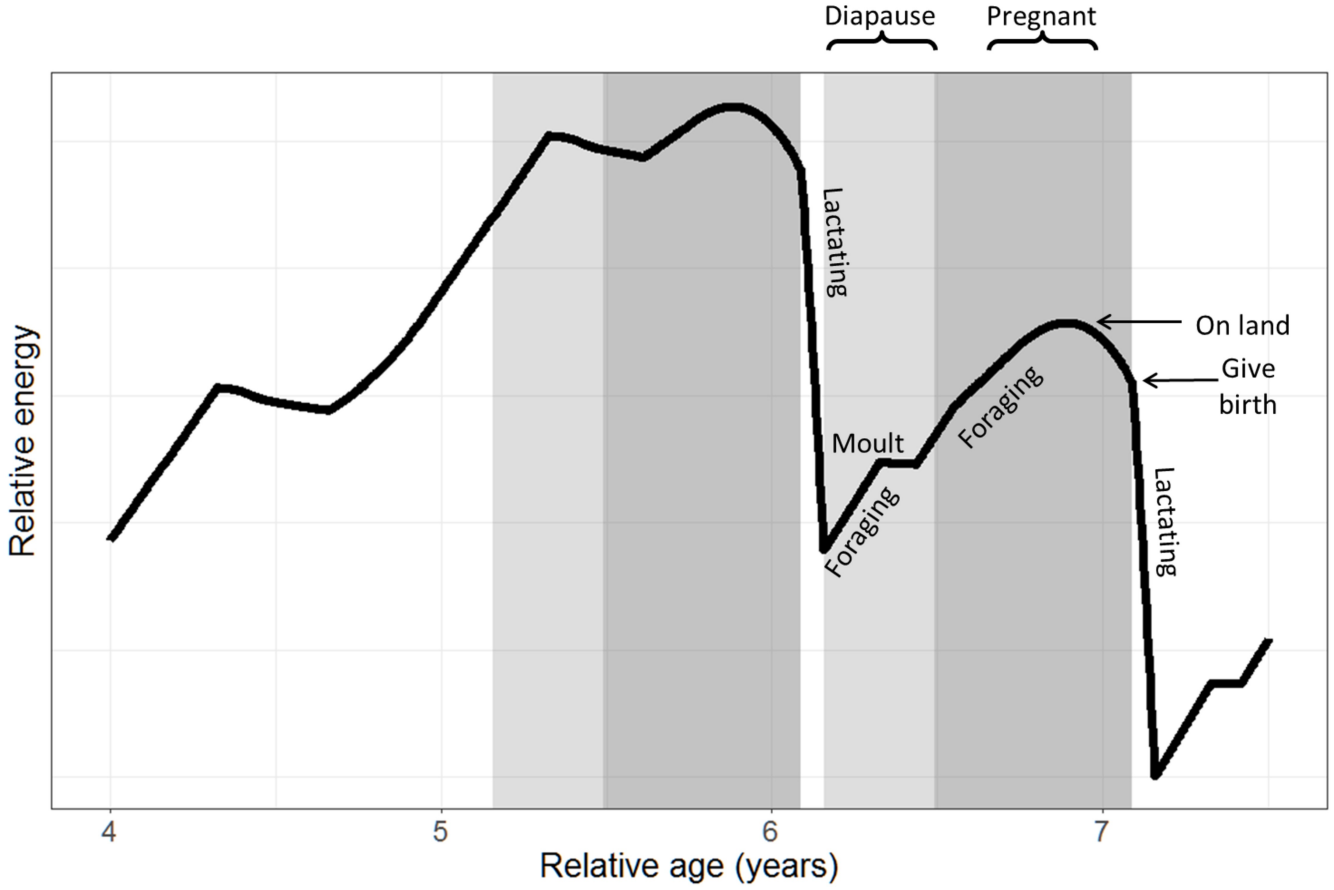
Model results for relative energy storage and use by an individual mother over two consecutive pregnancies. Model results for relative energy storage and use by an individual mother over two consecutive pregnancies. Relative age is included to show the timeframe before, during, and after pregnancies. Energy stores are depleted during fasting periods while the individual is on land (moulting and lactating), and are replenished during foraging trips (pre- and post-moulting periods). The reproductive energy storage *U*_*R*_ fluctuates with behaviour, and pregnancy requirements (as labelled). Grey background panels indicate stages of pregnancy: light grey indicates the period from conception to implantation (120 day diapause [48]); and dark grey indicates post-implantation (*i.e*. foetal development) period.

Female southern elephant seals breed between September and November and are impregnated while suckling their pup [36]. The pregnancy lasts for approximately 217 days, with implantation of the blastocyst delayed until February the following year, after the annual moult [48]. While on land, and suckling her pup, the mother fasts for around 30 days [48]. After weaning, the mothers return to the sea to replenish the energy they have lost [49]. They return for a moulting period, 60-70 days later, in January [36, 47, 50]. Fig 2 provides a detailed schematic of the relative energy use of a breeding female. Males arrive on land prior to the female’s breeding period, and can stay there up to three months between early August and late October, depending on their success in gaining a harem. They then return for a moulting period between February and late April depending on age (older bulls first). Adult males are not seen on the island in winter [36].

The pups weigh around 45 kg at birth, and 117 kg when they wean 23 days later (*e.g*. [51, 52, 53]). This rapid weight gain is possible due to the extreme ‘fattiness’ of southern elephant seal milk (16.1±6.98% fat on day one, up to 39.5±15.2% fat at weaning [54]). After weaning the pups stay on land for 4-5 weeks [36] before going to sea to forage [36]. In winter, the juveniles return to land for a mid-year haul-out [47, 55].

#### Model details

Dynamic energy budget theory characterises individuals through descriptions of their *structure, reserves, maturity*, and *reproduction buffer*. *Structure* determines size, feeding rates and maintenance costs. *Reserves* account for energy storage, which is utilised following the kappa-rule [18]. The kappa-rule (Fig 1) states that absolute priority is given to the energy allocation for growth and somatic maintenance (*κ*). The remaining energy (1 − *κ*) is allocated to maturity, maturity maintenance, and reproduction. *Maturity* is a continuous state variable that regulates transition between stages at fixed levels. Here we use foetal, pup, juvenile, and adult stages to represent the seals, with transition parameters at birth, weaning and puberty, where puberty indicates the transition threshold between juvenile and adult stage and is solely reliant on the individual’s energy storage, regardless of age or breeding status. *Reproduction buffer* is the energy stored for reproduction which is allocated to foetal and pup growth by pregnant or lactating individuals. The specific DEB rules regarding homeostasis and thermodynamics [18] are covered in this DEB-IBM through utilisation of DEBtool for the collection of DEB state variables.

The DEB-IBM follows energy levels and behaviour of individual female southern elephant seals through their full life cycle, from conception to death. The start of the model requires a run-in period of approximately 50 years to allow for emergent properties of individuals to settle and for the model to reach a stable population. This run-in period allows for the ‘first generation’ seals to live, breed and die; the next generations start from conception, rather than estimated initialisation values, and become emergent model components. For simplicity, in the model set-up (see section Initialisation in Model description, below), 250 individuals are created, none of which start out pregnant or with offspring. The number of females with pregnancies or offspring thus becomes an emergent feature dependent on the levels of the mother’s reproductive buffer.

**Fig 3:**
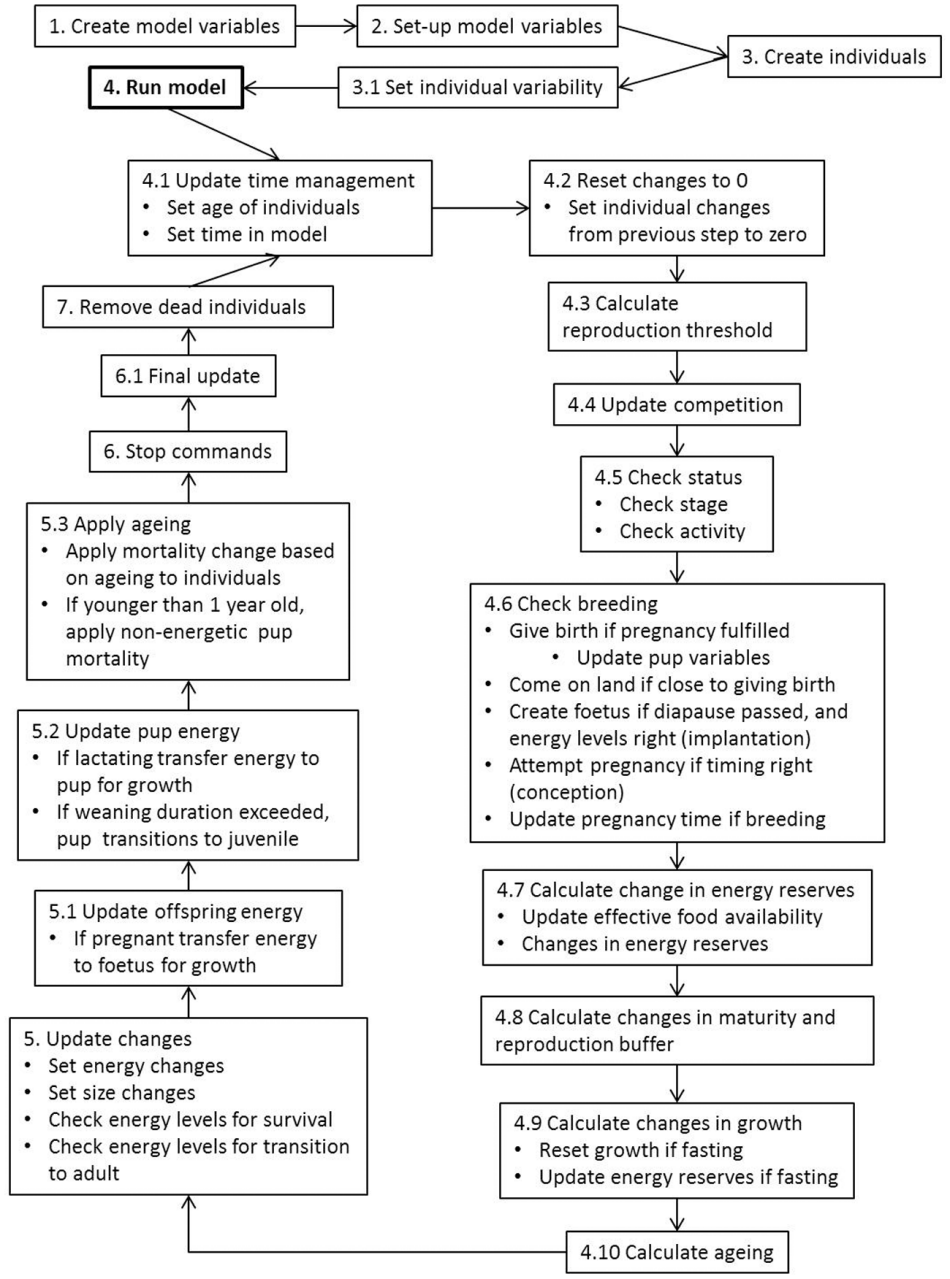
Model process of DEB-IBM. The set-up process of the model (steps 1-3), followed by the daily process (*i.e*. at each time-step) of the DEB-IBM for southern elephant seals (steps 4-7). Headings of steps 4-7 follow the headings of the sub-models as described in the ODD (Overview, Design concepts and Details) in section Sub-models in Model description.

The model runs on daily time-steps over a year, for a user-defined duration. We have used a 360 day annual cycle (*i.e*. each month consist of 30 days) as, considering the model does not include in-depth weather or other natural events, this approximation simplifies the model significantly. This modification also eliminates the need for implementation of leap years, which would add considerable complexity to the time frame of the individual based model. To account for a shorter year, we have modified the life cycle of southern elephant seals accordingly (including breeding, weaning and moulting times). Each seal still only breeds once per year and fasting, moulting and foraging occur at appropriate annual cycles. After set-up of the model, the ‘daily’ model process is applied as follows (Fig 3, and see section Sub-models in Model description, below for more details): i) as the date in the model is updated, each individual ages one day; the previous day’s changes are reset to zero and the competition for food is recalculated based on the potential new population numbers, ii) each independent individual (those not reliant on their mother; thus excluding foetuses and pups) checks their activity and breeding status, calculates their changes in reserves, maturity or reproductive buffer, and length, and calculates their physical aging (due to the accumulation of damage inducing compounds (see [18])), iii) the calculated changes are implemented and energy levels are checked for survival, iv) juveniles transition to the next stage if energy levels permit, v) pregnant mothers update their foetus’ variables, vi) nursing pups calculate their changes in reserves, maturity and length, and update their variables and the reproductive buffers of lactating mothers are updated again (if pups have reached their energy threshold, they transition to juvenile stage), vii) all individuals apply age related mortalities for old age, or non-energetic mortality for yearlings (see section Sub-models in Model description, below) viii) the model output is updated, and dead individuals are removed before the next time step begins.

### Model description

The model description follows the ODD (Overview, Design concepts and Details) protocol for describing individual-based models [56, 57]. The DEB-IBM was developed in the open-source agent-based modelling framework NetLogo (version 6.0.1 March 2017; [58]) for female southern elephant seals. We modified the scaled (see section Model modifications) framework built by Martin *et al*. [16] for water fleas (NetLogo version 4.1.1, 2010) to fit southern elephant seals.

We follow the DEB notations for parameters, as per [16, 18, 59]; a full list of parameters used for this model can be found in Tables 1 and 2; published parameters for southern elephant seals from Macquarie Island were used for parameter settings of both the DEB and IBM components of the model.

**Table 1:** DEB parameters and state variables used in the baseline model initialisation. All parameters follow DEB notations. ‘Entities’ refer to the entities in the model that are impacted by the parameters; “G” for global, “I” for individuals. ‘Change frequency’ indicates how often the parameter changes; “-” indicates no change. ‘Notes’ hold references for values relating to the population dynamics of southern elephant seals, relevant equations for parameters, and further details on parameters: where a = DEBtool value; b = user defined during the model development stage; c = emergent model value.

DEB not.	Value	Units	Discriptor	Entities	Change freq.	Notes
*cv*	0.05	-	Initial individual variability	I	-	b
*iv*	-	-	Effective individual variability	I	At setup	eq 1
*g*	0.7138	-	Energy investment ratio	I	At setup	a
$\dot{\nu }$	0.065	cm d^-1^	Energy conductance	I	-	a
${\dot{k}}_{M}$	0.0014	d^-1^	Somatic maintenance coefficient	I	-	a
${\dot{k}}_{J}$	0.002	d^-1^	Maturity maintenance coefficient	I	-	a
*κ*	0.74		Fraction of mobilised energy to soma (*κ*), foetal development (*κ*_*F*_), and lactation (*κ*_*L*_)	I	-	a
*κ*_*F*_	0.725					b
*κ*_*L*_	0.02					b
*f*_a_	0.935	-	Initial food availability	G	-	b
*P*	-	Individuals	Population	G	Daily	c
*K*	1000	Individuals	Carrying capacity	G	-	b
${\dot{p}}_{Am}$	968.2785	J d^-1^ m^-2^	Surface-area-specific maximum assimilation rate	G	-	a
*δ*_*M*_	0.235		Shape coefficient	I	-	b
*f*_eff_	-	-	Effective food availability	I	Daily	eq 2
Δ*P*	-	-	Competition	I	Daily	eq 3
*L*	-		Volumetric structural length	I	Daily	eqs 4 and 18
*L*_*b*_	110	cm	Volumetric structural length at birth (b), weaning (x), puberty (p), and maturity (m)	I	-	[51, 52, 53]
*L*_*x*_	125					
*L*_*p*_	180					
*L*_*m*_	280					
*l*	-		Scaled structural length	I	Daily	eq 6
$E_{H}^{b}$	2.81×10^7^	J	Maturity threshold at birth (b), weaning (x), and puberty (p)	I	-	a
$E_{H}^{x}$	6.50×10^7^					b
$E_{H}^{p}$	1.45×10^8^					a
*e*	-	-	Scaled reserves per unit of structure	I	Daily	eq 8
$U_{H}^{b}$	2.90×10^4^	-	Scaled maturity thresholds at birth (b), weaning (x), and puberty (p)	I	-	eq 9
$U_{H}^{x}$	6.71×10^4^					
$U_{H}^{p}$	1.50×10^5^					
*U*_*E*_	-	-	Scaled reserve	I	Daily	c, following eq 5
*U*_*H*_	-	-	Scaled maturity	I	Daily	c, following eq 9
*U*_cum_	-	-	Cumulative energy req. for breeding	I	Annually	eq 10
*U*_*R*_	-	-	Scaled reproductive buffer	I	Daily	c, following eq 20
*S*_*C*_	-	-	Mobilisation flux	I	Daily	eqs 11 and 12
*S*_*A*_	-	-	Assimilation flux	I	Daily	eqs 13 and 14
${\ddot{h}}_{a}$	6.0×10^−10^	d^-2^	Weibull ageing acceleration	I	-	a
*s*_*G*_	0.1	-	Gompertz stress coefficient	I	-	a
$\ddot{q}$	-	d^-2^	Ageing acceleration	I	Daily	eq 21
$\dot{h}$	-	d^-1^	Hazard rate	I	Daily	eq 22

**Table 2:** IBM parameters as used in model initialisation. ‘Notes’ hold relevant equations for, and further details on, parameters: where a = user defined value; b = means taken and individual variability applied; eq 1; and c = value adjusted to fit 360 day model (see text).

IBM parameters	Value	Units	References	Notes
Individuals created at start of model	250	Individuals		a
Moult duration				
Pups	50	d		
Juveniles	26	d	[36, 50]	a, b, c
Adults	30	d		
Forage duration				
Juveniles	45	d		a, b, c
Adults	98	d		
Mid-winter haul-out for juveniles	15	d	[47]	a, b, c
Resting duration				
Juveniles	2	d		a,b
Adults	1	d		
Diapause	120	d		
Breeding duration	217	d	[48]	c
Weaning duration	23	d		
Chance of breeding failure for				
>3 year old	0.98			
>4 year old	0.21	-	[46, 60]	a, b
>5 year old	0.15			
>6 year old	0.75			
Non-energetic pup survival	65.96	%	[51, 55, 61]	a, eq 34

#### Purpose

The purpose of this model is to provide a basic framework to represent higher trophic-level predators with complex life histories in a detailed fashion. The model includes detailed representations of energy requirements and use, as well as (breeding) behaviour.

#### Entities, state variables, and scales

For the development of the model, the DEB parameters were collected using the ‘DEBtool’ toolbox for Matlab (version R2014a 8.3.0.532; http://www.debtheory.org/; latest version downloaded on 19-07-2016) to determine the state variables (defined by Kooijman [18] as a “variable which determines, together with other state variables, the behaviour of a system. The crux of the concept is that the collection of state variables, together with the input, determines the behaviour of the system completely.”) needed for simulation of the species’ life-cycle.

The model follows the scaled DEB-IBM of Martin *et al*. [16], with DEB parameters derived from DEBtool (see also section Model modifications, below) using input data—either user defined, or from the DEBtool database ‘add_my_pet’ (http://www.debtheory.org/). The DEB-IBM includes two entities; individuals (here, seals), and their environment. Individuals are represented using a number of DEB state variables as described in Table 1 for more details).

**Fig 4:**
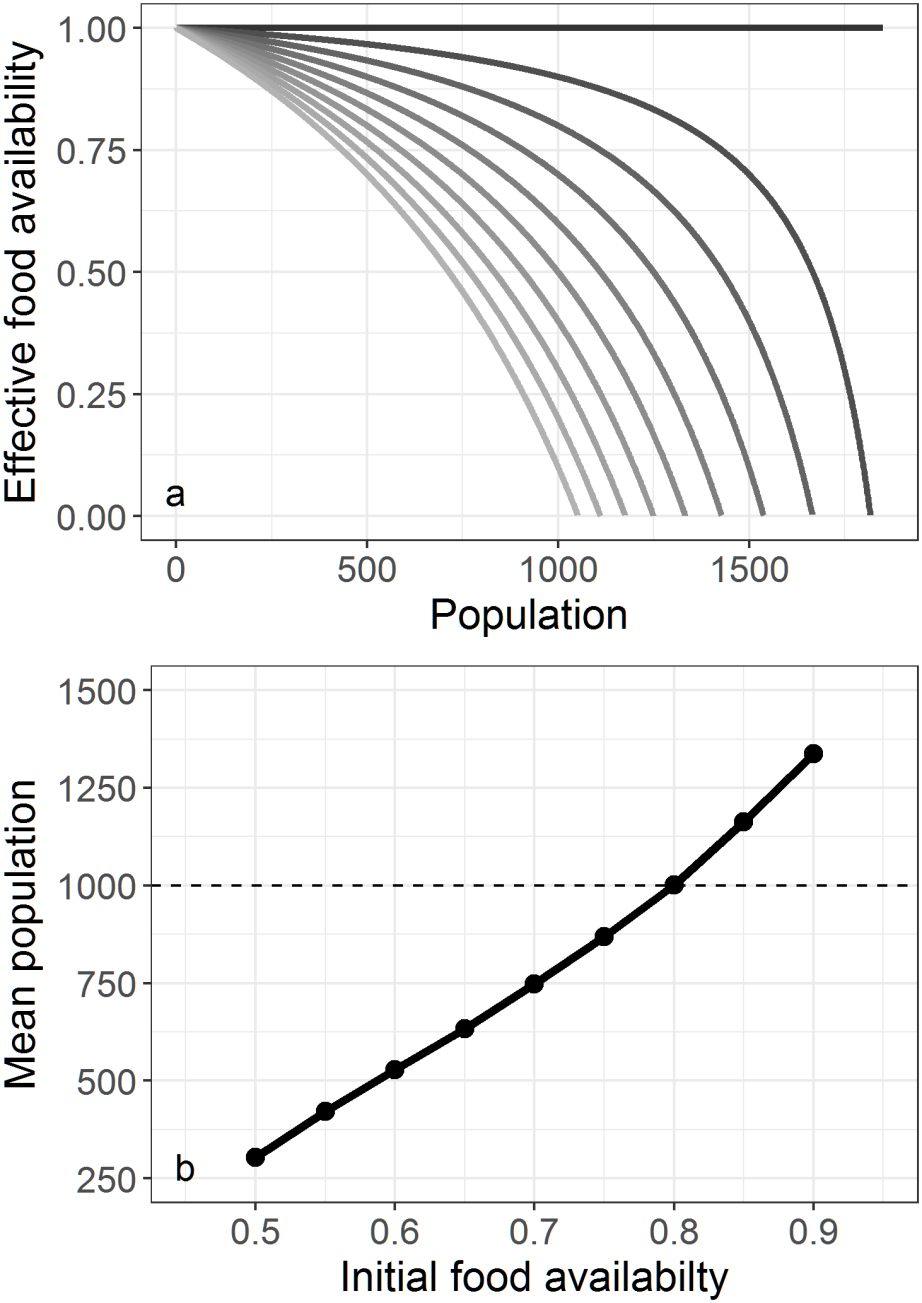
Comparison of initial and effective food availability at a range of population sizes. a) Theoretical effective food availability *f*_eff_ at different starting levels of *f*_a_ (range 0.1-1.00 at steps of 0.1: light grey to black) for a range of population *P* sizes, using eqs 2 and 3 for effective food availability with competition; assuming the individual variability is non-existent (*i.e*. *cv* = 0) and the carrying capacity *K* = 1000. The effective food availability is large for all starting conditions, when there are few individuals within the modelled population. As the population increases, the lower food availability (light grey) is most sensitive to change in population numbers. b) The model maintains a stable population over time, relative to the set *f*_a_ (0.50-0.95); whereas the population collapses with an initial food availability <0.50. Dotted line represents the carrying capacity *K* = 1000 individuals. Total mean of population shows only independent individuals (*i.e*. juveniles and adults) who are impacted by the competition for food (see section Initialisation in Model description).

The environment of the individuals is non-spatial and is represented by a set initial food availability *f*_a_ (dimensionless, range 0-1.00 representing 0-100% of food availability) which through the included competition term (see section Initialisation, below) becomes the effective food availability *f*_eff_ (see eq 2, and Fig 4). Time in the model is represented using finite difference equations for daily time-steps. The value for initial food availability *f*_a_ (Table 1) was modified from the value of 1.00 derived using DEBtool, as, although this gave accurate results for the remaining DEBtool derived parameters, this value was too high for the DEB-IBM we developed. An initial food availability of 1.00 assumes that there is unlimited food available, which is not the case for southern elephant seals (as they need to actively forage for their resources). Initial investigations in the model development stage determined that a value of 0.935 was the maximum value of *f*_a_ that resulted in a stable population. Values higher than this invariably led to an ever increasing population of seals.

#### Process overview and scheduling

Individual variables are updated every time-step, based on sets of finite difference equations. Discrete events, such as birth and death may occur based on the outcomes of these equations. Fig 3 describes a single time-step assuming that the initial set-up has already taken place. An independent individual is one that is no longer reliant on its mother (thus excluding foetuses and pups).

#### Design concepts

**Basic principles** The model is adapted from a DEB-IBM for water fleas developed by Martin *et al*. [16]. DEB theory [18] considers assimilation and energy use of individuals for growth, maintenance and reproduction via a balanced approach for mass and energy [16, 18]. This model thus follows the basic principles of DEB theory as well as IBMs.

**Emergence** Individual results, and consequent population dynamics, emerge through properties of metabolic organisation (DEB theory) and interaction between individuals (IBM).

**Adaptation** Adaptive behaviour is not included in the model. Individual variability is applied in the set-up of the model; however, over their lifespan the standard variables remain constant. Consequently the design concepts “Objectives”, “Learning”, “Prediction”, and “Sensing” do not apply in this model. This can change if spatial components are implemented in the model. Additionally “Collectives” are not represented in the model as each individual represents an individual.

**Interaction** Assumptions are made for interactions during the breeding season which allows females to become pregnant in a model which does not (currently) include males. Indirect interactions are included in the model through a competition formula affecting individual food availability.

**Stochasticity** Initial stochasticity is included in the model through individual variability *iv* from the initial parameter settings (see eq 1, and section Initialisation, below). Stochasticity is also included in the initialization of the individuals through a randomly calculated size *L* within the limits of juvenile (min) and adults (max); through ageing (mortality probability) and non-energetic pup mortality; and breeding sub-models (probability of failed mating at different age classes).

**Observation** A number of outputs are displayed on the user interface, and each can be exported with ease (see the NetLogo User Manual [58]). In the published model the output includes the overall population trends over time; as well as the population and stage class densities; growth; age at first reproduction and transition to puberty; mean ages, as well as lifespan and mortality causes; fecundity and pup survival. Ultimately, any individual or population based variable can be observed quite easily.

#### Initialisation

The initialisation of the model uses initial values for all parameters, as listed in Tables 1 and 2, unless otherwise specified below. The following describes the calculations made to implement individual variability to the model.

Individual variation *iv* (eq 1) is implemented in the model using the parameter *cv* for energy investment ratio *g* and effective food availability *f*_eff_ (eq 2), as well as for foraging, moulting, and resting durations. This individual variability is calculated with eq 1.

$$\begin{array}{c} iv=e^{\left(\text{random-normal}\;0\;cv\right)} \end{array}$$ (1)

This creates a log-normally distributed random number with a standard deviation which is user defined. Where “random-normal” is a NetLogo defined variable that reports a normally distributed random floating point number with a mean of 0 and a standard deviation of *cv* [58], (here, 0.05; Table 1).

The model includes a population-dependent competition term Δ*P* (eq 3) that directly influences each individual’s effective food availability *f*_eff_ (eq 2) through a scaling of the overall food availability term *f*_a_;

$$\begin{array}{c} f_{\text{eff}}=\left(f_{a}+\Delta P\right)iv \end{array}$$ (2)

where

$$\begin{array}{c} \Delta P=\left(1-f_{a}\right)\left(1-\frac{P}{2K-P}\right) \end{array}$$ (3)

If the overall food availability *f*_a_ is set so that the population *P* is stable, we require Δ*P* to be positive for a population that is less than the implemented carrying capacity (or expected equilibrium) *K*, approaching a maximum value of (1 − *f*_a_) as *P* becomes very small (and so effective food availability tends to 1). Once the population grows to a value above *K*, Δ*P* turns negative and decreases effective food availability (Fig 4a).

The form of Δ*P* is such that the penalty in effective food availability for increased population increases asymptotically as *P* approaches a population of 2*K*. The whole population is used to determine *P* and Δ*P* as dependent individuals (those reliant on their mother) make up <10% of the whole population, although only juveniles (including yearlings) and adults are used for analyses of population stability.

The inclusion of a competition term applies self-limitation to the population—if the population is larger than the point where crowding begins to limit the food available for each individual (*i.e*. *P* > *K*) then the food availability reduces proportionally. Variability in individual fitness and performance is implemented through a random variation in the effective food availability (see eq 2).

At the initialisation of the model, the length *L* (eq 4) of each individual is set at a random value between the length at weaning *L*_*x*_ and ultimate length *L*_*m*_, and is multiplied by the shape coefficient *δ*_*M*_ to convert physical length (in cm) to a dimensionless structural length (see [18]):

$$\begin{array}{c} L=L_{x}+\text{random}\left(L_{m}-L_{x}\right)\;\delta_{M} \end{array}$$ (4)

Initial reserve settings *U*_*E*_ (eq 5) are based on the individual’s length *L* (eq 4, or structural length *L*^3^), scaled length *l* (eq 6), and energy conductance $\dot{\nu }$

$$\begin{array}{c} U_{E}=L^{3}\;\frac{l}{\dot{\nu }\;} \end{array}$$ (5)

where

$$\begin{array}{c} l=\frac{L}{L_{max}} \end{array}$$ (6)

and

$$\begin{array}{c} L_{max}=L_{m}\;\delta_{M} \end{array}$$ (7)

Scaled maturity *U*_*H*_ at the current state and length for initialisation is calculated through dividing the scaled reserve *U*_*E*_ by 2.87. This value is a mean calculated from maturity levels *U*_*H*_ over reserves *U*_*E*_ at lengths for weaning and puberty. This is slightly inaccurate, but only impacts the first individuals that are created in the model; *U*_*E*_ and *U*_*H*_ (as well as *U*_*R*_) become emergent features for the next generation of individuals (see section Design concepts, above).

Scaled reserve density *e* (eq 8) is calculated for the initialisation of the model, as well as at each time step with calculation of change in reserves. It represents the amount of reserves per unit of structure relative to the maximum amount of reserves per unit of structure (*i.e*. the available energy stored over a period of time, which is particularly important during periods of fasting [16, 18]):

$$\begin{array}{c} e=\dot{\nu }\;\frac{U_{E}}{L^{3}} \end{array}$$ (8)

A sanity check is performed here, to ensure no individuals have maturity levels lower than that of a young juvenile; *i.e*. the current *U*_*H*_ is compared with set threshold levels. These threshold levels ($U_{H}^{b},U_{H}^{x}$ and $U_{H}^{p}$, respectively for birth, weaning, and puberty; Table 1) represent the threshold values at which an individual transitions to the next stage (foetus, to pup, to juvenile, and ultimately to adult). These are calculated (eq 9) from $E_{H}^{b},E_{H}^{x}$ and $E_{H}^{p}$ as derived from DEBtool, using the surface-area-specific assimilation rate $\left\{{\dot{p}}_{Am}\right\}$ for the scaled model:

$$\begin{array}{c} U_{H}^{b}=\frac{E_{H}^{b}}{\left\{{\dot{p}}_{Am}\right\}} \end{array}$$ (9)

Based on the scaled maturity of the individual, their stage (juvenile or adult), age (between three and 15 years) and hazard rate $\dot{h}$ (eq 22; for ageing purposes) are set. Their scaled reproductive buffer *U*_*R*_ is set equal to the scaled maturity (this again is balanced out as an emergent feature over the next generation of seals).

The reproductive threshold *U*_cum_ (eq 10) is included in the model to control the number of births per individual, and is proportional to the individual’s size (see section Thresholds for puberty, breeding and death in Model modifications, below). This threshold is modified from Kooijman ([62], page 38), and considers the cumulative energy requirements for foetal development of southern elephant seals, proportional to the mother’s size:

$$U_{\text{cum}}={\left(L_{w}^{b}\;\delta_{M}\right)}^{3}\;\left(\frac{f_{\text{a}}+g}{\dot{\nu }}\right)\;\left(1+\frac{3}{4}\;\frac{L_{w}^{b}/L_{w}^{m}}{f_{\text{a}}}\right)\;0.2\;l$$ (10)

All remaining settings are set so that none of the individuals are pregnant or have mated, and all individuals are foraging. The model starts on the first of January and uses a run-in period of 50 years to allow for emergent features to come through and improve the stochasticity in the model.

#### Input data

The model does not use input data to represent time-varying processes. Tables 1 and 2 summarise the DEB and IBM parameters and the values as they are used in the model.

#### Sub-models

The following sub-models are implemented for all individuals (unless otherwise specified) that have not died in this time-step. The calculations for changes in energy reserves, maturity, reproductive buffer and growth follow formulations for the scaled model by Martin *et al*. [16], which are “algebraically rearranged, reduced (using compound parameters), and scaled with the aim of reducing the amount and types of data needed to parameterize the model for a species”. An in-depth guide has been provided by Martin *et al*. [16] in their user manual—which is applicable for the following sub-models, unless otherwise specified. Formulations and deviations used in this DEB-IBM for southern elephant seals are provided here.

**Update time management** The time management sub-model handles the timings of the model. Each time-step represents a single day. At each time-step a day is added to the year as well as to the month, and each individual adds a day to its age. At the end of each month (30 days) the days of the month are reset, and a month is added. When 360 days have passed, the day of the month, day of the year and month of the year are set back to 1 and a year is added to the count.

**Reset changes to 0** At each time-step each individuals clears changes set in previous time-steps. Thus *dU*_*E*_, *dU*_*H*_, *dU*_*R*_, and *dL* (see below) are set to 0.

**Calculate reproduction threshold** At the start of each time-step the individuals re-calculate their reproductive threshold (eq 10), as this is proportional to their size.

**Update competition** The effective food availability *f*_eff_ of individuals is updated to include the most recent change in competition, as per eqs 2 and 3.

**Check status** Each individual has a stage (foetus, pup, juvenile, mature; 0-3, respectively), and a status (mother-dependent, fasting, foraging; 0-2, respectively) which can change, logically, throughout its lifecycle. This sub-model handles the status of each independent individual (*i.e*. those not reliant on their mother).

- Maximum duration of moulting, resting and foraging are set according to their current stage and age.
- Then for the relevant status, a day is added to each ‘activity’ (foraging, fasting, resting, moulting); if days exceeds the maximum days set for the activity, the activity is changed (*i.e*. from fasting to foraging).
- If the month is December (12) and they are not yearlings (age <360 d), individuals start their annual moulting process.
- If the month is July (7) the juveniles start their annual mid-winter-haul-out.

**Check breeding** This sub-model handles the breeding process—this, when activated, uses additional sub-models. The breeding checks are processed in reverse-chronological order (from giving birth to impregnation of the mother) so that each action is handled in a subsequent time-step (*i.e*. it is not possible to add a day to a pregnancy and then in the next section already give birth).

a If the total time of pregnancy has been reached (*i.e*. the time since breeding = total breeding duration + diapause) the individual gives birth. Here settings are altered so that the mother is resting and lactating, but no longer pregnant or impregnated. Here total number of offspring over her lifetime is updated and the age and stage of the offspring are updated.
b If the mother is 8 days from giving birth (*i.e*. the time since breeding = total breeding duration + diapause - 8 days) her settings are updated so that she comes on to land (status = fasting) in preparation for birth (as per [48, 63, 64]).
c If the time since mating equals the species’ diapause duration the pregnancy is implemented. The first check is then to make sure that the individual has enough energy to support a foetus through to birth (*i.e*. *U*_*R*_ higher than the reproductive threshold as calculated in eq 10). If these energy levels aren’t reached, then pregnancy is aborted and the individual continues foraging. If pregnancy occurs: a new offspring is ‘hatched’; individual variables are implemented; and the two individuals are connected via their respective IDs.
d If the individual has been impregnated, or is pregnant, a day is added to her pregnancy.
e If the individual is breeding but not yet pregnant or impregnated, impregnation happens. No new individuals are created here as diapause has not yet passed. Rates of successful impregnation depend on the age of the individual (see Table 2). So long as they are within a reasonable number of pregnancy attempts (this is set here to a 7 day period), they can try again in the next time-step.
f If individuals are lactating and have been on land for 19 days [48] they are ready for their next pregnancy. The impregnation sub-model is then implemented.

As the model follows an actual population and lifecycle, the months of year for breeding are important. Offspring are born sometime at the end of September, beginning of October and thus the modelled breeding cycle needs to follow this.

g If the month is September (9) individuals check that they have enough energy for breeding and that they are old enough for breeding, as above.
h If all is good—breeding is implemented and the sub-models will be activated in the next time-step.
i If the month is November (11) and individuals are indicating they can breed, but have so far had no luck they are classified as failed breeders and will return to foraging.

**Calculate change in energy reserves *dU*_*E*_** The change in energy reserves is determined by the difference between the scaled mobilization *S*_*C*_ (eq 11 or 12) and assimilation *S*_*A*_ (eq 13 or 14) fluxes. The first step in the calculation for change in energy reserves is to ensure that the individual’s effective food availability includes the competition term (eq 3). If *f*_eff_ > 1 then *f*_eff_ is set to 1 as there cannot be more than 100% food availability. The scaled energy reserve *e* (eq 8) is recalculated.

The mobilisation flux *S*_*C*_ (eq 11) represents the energy used, following the calculation used by Martin *et al*. [16]

$$\begin{array}{c} S_{C}=L^{2}\;\frac{g\;e}{g+e}\;(1+\frac{L{\dot{k}}_{M}}{\dot{v}}) \end{array}$$ (11)

If the individual is in fasting mode (due to resting or moulting) there is no food intake, and *f*_eff_ is set to 0; the mobilisation flux (eq 12) changes to

$$\begin{array}{c} S_{C}=\frac{{\dot{k}}_{M}\;g\;\kappa }{\dot{v}}\;L^{3} \end{array}$$ (12)

The assimilation flux *S*_*A*_ (eq 13) represents the consumption of food proportional to the surface area, following the calculation as per Martin *et al*. [16]

$$\begin{array}{c} S_{A}=f_{\text{eff}}\;L^{2} \end{array}$$ (13)

If the individual is pregnant, up-regulation takes place [18] and the surface area of the foetus *L*_*foetus*_ is included in the assimilation flux (*S*_*A*_; eq 14), thus

$$\begin{array}{c} S_{A}=f_{\text{eff}}\;(L^{2}+L_{foetus}^{2}) \end{array}$$ (14)

If the individual is a yearling and foraging, an 80% chance is implemented that they are less successful at finding food, and will thus only collect 20% of their otherwise effective food available. The final calculation is for the collection of actual stored energy *dU*_*E*_ (eq 15), assuming energy has already been used through the mobilisation flux *S*_*C*_ (eq 11 or 12),

$$\begin{array}{c} dU_{E}=S_{A}-S_{C} \end{array}$$ (15)

**Calculate change in maturity *dU*_*H*_ and reproduction buffer *dU*_*R*_** Independent seals need to calculate their change in maturity levels and/or reproduction buffer. Juvenile individuals (with $U_{H}<U_{H}^{p}$) calculate their change in maturity *dU*_*H*_ (eq 16):

$$\begin{array}{c} dU_{H}=\left(1-\kappa \right)\;S_{C}-{\dot{k}}_{J}\;U_{H} \end{array}$$ (16)

where ${\dot{k}}_{J}\;U_{H}$ represents the maintenance cost associated with maintaining their current levels of maturity (${\dot{k}}_{J}$ = maturity maintenance rate coefficient). *S*_*C*_ is as per eq 11 or 12; whichever is relevant to the individual’s foraging status.

If individuals have reached the maturity threshold and are considered adults, they calculate the change in their reproductive buffer (eq 17). This is calculated as per the change in maturity (eq 16), but uses the maximum level of maturity maintenance required by adults $U_{H}^{p}$

$$\begin{array}{c} dU_{R}=\left(1-\kappa \right)S_{C}-{\dot{k}}_{J}\;U_{H}^{p} \end{array}$$ (17)

**Calculate growth *dL*** Growth, or change in structural length (eq 18), is calculated for individuals who have not yet reached maximum size (i.e. *L* < *L*_*M*_)

$$\begin{array}{c} dL=\frac{1}{3}(\frac{\dot{v}}{g\;L^{2}}\;S_{C}-{\dot{k}}_{M}L) \end{array}$$ (18)

In the case where scaled reserve density *e* (eq 8) falls below the scaled length *l* (eq 6) there is not enough energy to sustain growth [16] and *dL* is set to 0 while the starvation mode is implemented (see eq 12). Consequently, energy is diverted from growth to pay for somatic maintenance ${\dot{k}}_{M}$ and thus the original calculations for maturity and reproductive buffer (eqs 16 and 17) are replaced with

$$\begin{array}{c} dU_{H}=\left(1-\kappa \right)S_{C}-{\dot{k}}_{J}\;U_{H}^{p}-\kappa L^{2}\left(l-e\right) \end{array}$$ (19)

and

$$\begin{array}{c} dU_{R}=\left(1-\kappa \right)S_{C}-{\dot{k}}_{J}\;U_{H}^{p}-\kappa L^{2}\left(l-e\right) \end{array}$$ (20)

respectively, using the maximum value for *U*_*H*_ ($U_{H}^{p}$) for maintenance allocation for both calculations. As there has been a change in the mobilisation flux *S*_*C*_ (eq 12) the scaled energy reserve *dU*_*E*_ is recalculated as per eq 15. If the scaled reserve density *e* ≤ 0 the individual dies, and links, where relevant, are broken between mother and offspring.

**Calculate ageing** The ageing sub-model (see [16, 18]) is applied to all individuals from the day that they are born and is applied as a deterioration of structure over time using the DEB parameters ageing acceleration $\ddot{q}$; Weibull ageing acceleration ${\ddot{h}}_{a}$; and hazard rate $\dot{h}$ [18]. Ageing is assumed to occur due to accumulation of damage inducing compounds proportional to the mobilisation flux *S*_*C*_. The cumulative scaled acceleration (eq 21) and hazard (eq 22) rates are calculated for implementation in the ageing sub-model:

$$\begin{array}{c} \ddot{q}=\ddot{q}+d\ddot{q} \end{array}$$ (21)

$$\begin{array}{c} \dot{h}=\dot{h}+d\dot{h} \end{array}$$ (22)

where $d\ddot{q}$ (eq 23), and scaled hazard rate $d\dot{h}$ (eq 24), are as per Kooijman ([18], page 216)

$$\begin{array}{c} d\ddot{q}=((\ddot{q}\frac{L^{3}}{L_{M}^{3}}\;S_{C}+{\ddot{h}}_{a})e(\frac{\dot{v}}{L})-\dot{r}\ddot{q}) \end{array}$$ (23)

$$\begin{array}{c} d\dot{h}=\ddot{q}-\dot{r}\dot{h} \end{array}$$ (24)

where $\dot{r}$ (eq 25) is the rate of growth

$$\begin{array}{c} \dot{r}=\frac{3}{L}dL \end{array}$$ (25)

**Update changes** For female southern elephant seals breeding can start at the age of three, whereas somatic growth continues until the age of six [46]. The calculations for *dU*_*R*_ (eq 20) are, however, only carried out for adults and thus remain at 0 for individuals who are yet to reach maturity. To accommodate for allocation of energy to reproduction while the individual is yet to reach maturity, for these individuals *dU*_*H*_ is split on a 60: 40 ratio between *dU*_*R*_ and *dU*_*H*_ (based on trials during the model development stage).

As all the calculations have been carried out, changes for *dU*_*E*_, *dU*_*H*_, *dU*_*R*_, and *dL* need to be implemented through the simple addition of *U*_*E*_ = *U*_*E*_ + *dU*_*E*_; and the same for the remaining changes. Where the accumulated *U*_*H*_ of juveniles exceeds their transition limit $U_{H}^{p}$ the remainder of *dU*_*H*_ is transferred to their reproductive buffer *U*_*R*_.

Yearlings have a lower survival rate than older individuals (which is related to their fitness and experience/success at foraging, as well as their mother’s fitness; *e.g*. [55]). To implement this in the model, an additional energetic related mortality check is added where if $U_{H}<0.92\times U_{H}^{x}$ the yearling dies. A sanity check is implemented here to ensure that individuals, whose energy levels have fallen below 0, die. This check also ensures that if a mother dies during a pregnancy, the foetus also dies. Connections are terminated if a mother or pup dies during the weaning stage, and the relevant variables are updated for the mother (or pup) who survives. If a mother dies while lactating, the pup goes into fasting mode until completion of the moulting period (∼50 days; Table 2).

If juvenile seals have reached puberty ($U_{H}\geq U_{H}^{p}$) they transition to adult stage. Changes from foetus to pup are handled in the breeding sub-model; changes from pup to juvenile are handled in the update offspring energy sub-model.

**Update offspring energy** The updating of offspring (foetus) energy is applied from a mother’s position. As the foetus is immobile, there is no mobilization flux used in any calculations, and the energy reserves are assumed equal to that of the mother [62]. The first step is to update the foetus’ growth (eq 26)

$$\begin{array}{c} L=L+r_{B}L_{M} \end{array}$$ (26)

using the von Bertalnaffy growth rate *r*_*B*_ (eq 27)

$$\begin{array}{c} r_{B}=\frac{\dot{v}}{0.545\;L_{M}} \end{array}$$ (27)

which has been modified from the originally published $r_{B}=\dot{v}/\left(3\;f_{\text{eff}}\;L_{M}\right)$ [62], as when using the original equation, pregnancies lasted for 900 days and pups were too large (see section Model modifications, below). This is followed by the calculation for scaled energy reserves (eq 28)

$$\begin{array}{c} dU_{E}=e_{\text{mother}}\;L^{2}\;\kappa_{F} \end{array}$$ (28)

where the scaled energy reserves of the mother *e*_mother_ are used for the calculation of energy uptake from food, proportional to the foetus’ surface area and the increased assimilation capabilities *κ*_*F*_. In case of foetal development, all energy reserves are used to reach maturity and thus the scaled maturity equals the scaled energy reserves, thus *dU*_*H*_ = *dU*_*E*_. The changes are then implemented following the simple addition of *U*_*H*_ = *U*_*H*_ + *dU*_*H*_ for *U*_*H*_ and *U*_*E*_. The mother’s reproductive buffer *U*_*R*_ is updated through the removal of the energy allocated to the foetus

$$\begin{array}{c} U_{R}=U_{R}-\left(dU_{E\;\text{foetus}}\;\kappa_{F}\right) \end{array}$$ (29)

**Update pup energy** A sanity check is performed to ensure the pup has a mother, following which the calculation for scaled energy reserve *dU*_*E*_ is as per eq 15, where the assimilation flux *S*_*A*_ changes (eq 30)

$$\begin{array}{c} S_{A}=\frac{f_{\text{eff}}L^{2}}{\kappa_{L}} \end{array}$$ (30)

The effective food availability *f*_eff_ is set to 2 × *f*_a_, and *κ*_*L*_ is implemented to allow for the increased ‘fattiness’ of southern elephant seal milk (up to 55%; see Hindell *et al*. [54]) as well as the increased allocation efficiency of milk. The mobilisation flux *S*_*C*_ for pups (eq 31) becomes

$$\begin{array}{c} S_{C}=3(L^{2}\frac{g\;e}{g+e}(1+\frac{L{\dot{k}}_{M}}{\dot{v}})) \end{array}$$ (31)

Calculations for scaled maturity *U*_*H*_ are as per eq 16, and *dU*_*R*_ = 0. The change in growth *dL* of pups (eq 32) is modified from eq 18 to account for the increased growth rates of southern elephant seals during weaning

$$\begin{array}{c} dL=\frac{\dot{v}}{g\;L^{2}}\;S_{C}-{\dot{k}}_{M}L \end{array}$$ (32)

The calculated changes are applied to the pup, and the energy allocated by the mother are removed from her reproductive buffer (eq 33)

$$\begin{array}{c} U_{R}=U_{R}-\left(S_{A\;\text{foetus}}\;\kappa_{L}\right) \end{array}$$ (33)

During the weaning period, the mother tracks the time that she has been lactating. Once this period exceeds the individual’s weaning duration, the link between mother and pup is broken, and the pup’s status is updated to juvenile. The pup remains on land for a moulting period while the mother returns to foraging.

**Apply aging** The ageing previously calculated is now applied to the scaled hazard rate $\dot{h}$ (eq 22) through a randomly selected range between 0 and a user-defined mortality variable (mortality-float) multiplied by the individual’s individual variability (eq 1). If the individual variability is less than 0.95, the mortality chance is increased to account for the lesser overall fitness of the individual. If the mortality value is less than the hazard rate $\dot{h}$, the individual dies and any links with offspring or mother are severed, unless the mother is pregnant when she dies—then the foetus also dies.

Non energetic pup mortality is also dealt with here for pups and yearlings. If a randomly selected value (between 0 and 1) is less than the value set for the pup mortality (see Table 2), the pup or yearling dies. The pup-mort parameter is set at a user defined variable ranging between the minimum and maximum observed pup mortality; following data collected by several authors (*e.g*. [51, 55, 61]) from Macquarie Island. The pup mortality (eq 34) is converted from annual chance of survival to daily chance of mortality using the scaling:

$$\begin{array}{c} \text{daily-pup-mort}=\frac{1}{360}\;(1-\frac{x}{100}) \end{array}$$ (34)

where *x* is the annual chance of survival (as a percentage) from the non-energetic pup survival as presented in Table 2).

**Stop commands** There are three stop commands applied to the model which are implemented when the model’s run time has passed the set time that the model is set to run (in years); when the population has collapsed (i.e. there are less than 20 individuals left in the model), and; when the population has exceeded 50 times the starting population (assuming a starting population of 250, this becomes 12,500), thus reducing computational limitations.

**Final update** The final update for the model includes collecting the final information from individuals who died in this time-step—as this information is needed for collection of results (maximum age, size and number of offspring). Once this last set of data has been stored, the output is updated according to user defined requirements (*e.g*. total count, population dynamics, fecundity of females, length of individuals, etc.).

**Remove dead individuals** The individuals who died in previous sub-models are now removed from the model. This is done as the final step so that all the information gained in the time-step can be collected before ‘dead’ individuals are removed from the model.

### Model modifications

We adopted the scaled version of the standard DEB model following Martin *et al*. [16], meaning that the model was simplified as the state variables for *reserves* *E*_*E*_, *maturity* *E*_*H*_, and the *reproduction buffer* *E*_*R*_ are divided by the maximum surface-area-specific assimilation rate $\left\{{\dot{p}}_{Am}\right\}$. This removes the units of energy from the model [16, 65]. This allows the use of scaled reserve *U*_*E*_, scaled maturity *U*_*H*_, and scaled reproduction buffer *U*_*R*_, as well as scaled life-stage transition parameters (threshold values) for birth $U_{H}^{b}$, weaning $U_{H}^{x}$, and puberty $U_{H}^{p}$; see Table 1.

#### Competition

The DEB-IBM for southern elephant seals is not spatially resolved. As such it cannot explicitly model the effects of overcrowding leading to increased competition for food and greater metabolic costs of longer foraging trips. To account for these limitations the model includes a population-dependent competition term Δ*P* (eq 3) that directly influences each individual’s effective food availability *f*_eff_ through a scaling of the overall food availability term *f*_a_ (eq 2) as explained in section Initialisation, above.

#### Foetus and pup development

Calculations for foetal and pup growth (eqs 26–33; section Sub-models, above) are based on Kooijman [18], but have been modified for this model, following Kooijman [62] and Roberts [30]. These modifications take into account the expected length and weight of pups at birth (110 cm and 45 kg) and weaning (125 cm and 117 kg; *e.g*. [51, 52, 53]), as well as pregnancy and weaning durations (217 and 23 days, respectively; [48]).

Although predicted weights and sizes from an initial model were similar to those observed for pups at Macquarie Island, both the pregnancy and weaning durations were too high in the model. Foetal growth in the model was too slow when using the original equation (von Bertalnaffy growth rate); pregnancies lasted around 900 days (expected 217 days [48]). To resolve this we adjusted the equation for foetal growth (eq 27) by reducing the impact that ultimate size and the mother’s effective food availability *f*_eff_ have on the growth rate. We also included the increased assimilation capabilities of the foetus *κ*_*F*_ to the energy transferred from the mother (eqs 28 and 29).

As the weaning duration in the model (269 days) was too high (expected 23 days [48]) we modified the equations for the pups’ energy intake and growth (eqs 30–33, section Sub-models). These changes take into account the short weaning period of southern elephant seals, the extreme weight gain of pups (∼70 kg between birth and weaning), and the extreme fattiness of southern elephant seal milk (up to 55% toward the end of weaning [54]). The species used for the development of these original equations by Roberts [30] were the tammar wallaby *Macropus eugenii* and echidna *Tachyglossus aculeatus*. The fat content in the milk for these species is much lower than that of the southern elephant seal; around 4% and 31%, respectively [66]. To account for this, we added a pup assimilation factor *κ*_*L*_ to eq 30 (for calculations of the scaled energy reserve *dU*_*E*_ and mobilisation flux *S*_*A*_) and 33 (the mothers’ reproductive energy *dU*_*R*_ expenditure) to increase the pups’ energy intake.

To take into account the increased energy mobilisation of pups we modified eq 31 by increasing the mobilisation flux *S*_*C*_ by a factor of three, compared to the original implementation of the equation for foraging independent individuals (eq 11). The outcome of this equation is implemented in the calculation for physical growth *dL* (eq 32) where the physical growth of pups is tripled (compared to the original calculation, eq 18) to ensure that the changes in growth are proportional to the changes in energy storage.

#### Yearling mortality

During the first 12 months, southern elephant seals have a higher mortality than for the rest of their life [67]. This is implemented in the model using two different methods; one for energetic mortality (starvation), and one for non-energetic mortality (*e.g*. predation by orcas *Orcina orca*).

Energetic mortality generally affects the yearlings soon after weaning as they are left on the beach by their mothers. In the first 4-5 weeks the yearlings go through starvation mode, after which they leave the island for the first time to forage. The pup mortality is larger for smaller seals (annual chances of survival vary between 71.6% for weaners heavier than 135 kg, to 54.2% for those weighing less than 95 kg; [51]). Although no conclusive data is available, it is expected that of the approximate 35% of yearlings that die, around 80% die of starvation, and 20% of non-energetic factors (Hindell, pers comm 2017). To account for this we have implemented a modified survival threshold in the model for yearlings, which is sensitive to reductions in stored energy. After initial results from model runs during development this threshold was set at 92% of their weaning threshold (which is directly linked to their mass). Additionally (see section Sub-models, above) a reduced chance of successful foraging has been implemented for yearlings, to account for their foraging naïvity.

Non-energetic mortality is presented in the model through a mortality parameter. The parameter is a user defined value between the minimum and maximum of observed yearling mortality, following data collected from Macquarie Island (*e.g*. [51, 55, 61]) and converted from annual chance of survival (field observations) to daily chance of mortality (modelled; see eq 34). The combination of the two mortalities balances out to the expected yearling survival rate.

#### Thresholds for puberty, breeding and death

The transition threshold from juvenile to adult stage $U_{H}^{p}$ has been changed from the DEBtool value to reduce the time it takes for an individual to become an adult. Using the original value, individuals transitioned to adult stage at around 15 years of age as opposed to the expected age of six [46].

As the population structure and projections in initial model development were particularly sensitive to changes in the reproductive buffer *U*_*R*_ of mothers, a breeding threshold *U*_cum_ was included in the model to set a minimum energy level at which individuals could sustain a pregnancy (eq 10). The inclusion of the breeding threshold allows for the exclusion of males in the DEB-IBM. This is validated based on the assumption that the population trajectory of southern elephant seals is only weakly dependent on male numbers (as explained in the introduction, as although there is a 1:1 ratio of females to males at birth, males make up only 36% of the adult population of which only ∼8% contribute to the next generation). Thus we added a breeding threshold *U*_cum_ which reduced the overall fecundity to near half of the observed fecundity in the field (up to 0.5 for female births by females [68]), and reduced the number of births over a lifetime below the expected breeding success (13 pups per lifetime [69]).

The reproductive buffer *U*_*R*_ contains the stores of energy solely for reproductive purposes (as opposed to maintenance and maturity). This buffer becomes depleted when a mother is pregnant, and particularly while she is lactating (as, during the final 30 days while she is on land, she does not take in any energy). The stored energy increases again following the pre- and post- moult foraging trips. If the stored energy exceeds the reproductive buffer *U*_cum_, the mother (if successfully impregnated) initiates her pregnancy after the diapause; if not then she aborts the pregnancy and skips that year of breeding. Thus, as the buffer is increased, it becomes more difficult to have consecutive pregnancies, particularly as mothers can lose up to 35% of their mass during lactation [60]. If the buffer is lower, more female seals are born and the population increases; when the buffer is set too low (below the cumulative cost of raising a pup) too many would-be mothers die during pregnancy, causing the population to collapse. At levels that were too high, too few pups were born as mothers chose not to breed, and again the population collapsed. The threshold (eq 10) is scaled to the size of the mother, as smaller mothers have less energy to allocate to foetal development [52].

During the model development stage, the sub-model for ageing was insufficient; individuals well exceeded their expected maximum age of 23 years. Consequently, a mortality parameter was included in combination with the DEB parameters to control the lifespan of individuals (see section Sub-models).

### Model evaluation and sensitivity analyses

The aim of the model evaluation was to determine the abilities and limitations of the model. For the southern elephant seal DEB-IBM this included i) being able to reproduce life histories as emergent model features, ii) being able to use the model to project a stable population over time iii) having realistic population dynamics and structure based on emergent life history features (such as age at first breeding, lifespan, fecundity and (yearling) survival).

We ran sensitivity analyses to test the limits of the model and to get a better understanding of the results the model might produce. The model we have built contains a large number of parameters, many of these are derived using DEBtool (see section Entities, state variables, and scales in Materials and methods, and Table 1, above) to ensure correct growth rates and energy intake and expenditure for the selected species. Of the remaining parameters those related to well observed characteristics of the species (such as life history traits and breeding behaviour) were not altered. For the sensitivity analyses we chose to focus on those parameters that directly influence the individual’s energy intake (initial food availability *f*_*a*_), and the required levels of stored energy for maintenance and maturity at which an individual transitions to the next stage of their life (transition thresholds at birth $U_{H}^{b}$, weaning $U_{H}^{x}$, and puberty $U_{H}^{p}$; Table 3). These thresholds are directly linked to each individual’s size and weight and therefore the levels at which these transition thresholds are set are expected to affect the lifetime success of the individual (and consequently of the population as a whole). The high (95%) and low (55%) values for the sensitivity analyses for food availability were chosen to represent extreme scenarios for resource availability that either makes the population grow excessively, or causes a near collapse of the population. The 10% change to the transition thresholds were chosen as indicative change representing our uncertainty in the parameters as these thresholds, specifically, represent the required size of the individuals at selected life stages (whereas other DEBtool derived parameters are used for the underlying mechanics).

**Table 3:** Parameter values used for sensitivity analyses. Parameter values used for baseline model and sensitivity analyses for changes in initial food availability *f*_a_ and transition thresholds for weaning $E_{H}^{x}$, and puberty $E_{H}^{p}$. Low and high variations for transition thresholds vary by 10% of the baseline value. ‘—’ indicates value is as per baseline model (*i.e*. no change).

	*f*_a_	$E_{H}^{x}$	$E_{H}^{p}$
Baseline	0.935	6.5 × 10^7^	1.45 × 10^8^
*f*_a_ low	0.55	—	—
*f*_a_ high	0.95	—	—
$E_{H}^{x}$ low	—	5.85 × 10^7^	—
$E_{H}^{x}$ high	—	7.15 × 10^7^	—
$E_{H}^{p}$ low	—	—	1.305 × 10^8^
$E_{H}^{p}$ high	—	—	1.595 × 10^8^

Given that we are using DEBtool for determining parameter values, using a greater than 10% variation of those DEB values takes us away from the theory of DEBtool; thus larger increases in the variation would discount theoretically derived values based on well tested general methods of DEB theory [18]. For example, the threshold for puberty $U_{H}^{p}$ was previously reduced in the model development stage (see section Thresholds for puberty, breeding and death in Model modifications, above) by close to 20% of the original value to match the complex life histories of southern elephant seals, indicating that changes of more than 10% could be unrealistic. We compared results of 10 Monte Carlo simulations of the sensitivity runs and analysed the results of 100 year simulations (after the run in period). Statistical analyses were done in *R* ([70], version 3.4.1, 2017) using two sided t-tests with a 99% confidence interval. The t-test is calculated using a sample size of 10, where each sample size is calculated as the mean of the 100 year run. The stable model, with the standard parameters is hereafter referred to as the ‘baseline model’.

## Results

### Population stability

#### Baseline model

**Fig 5:**
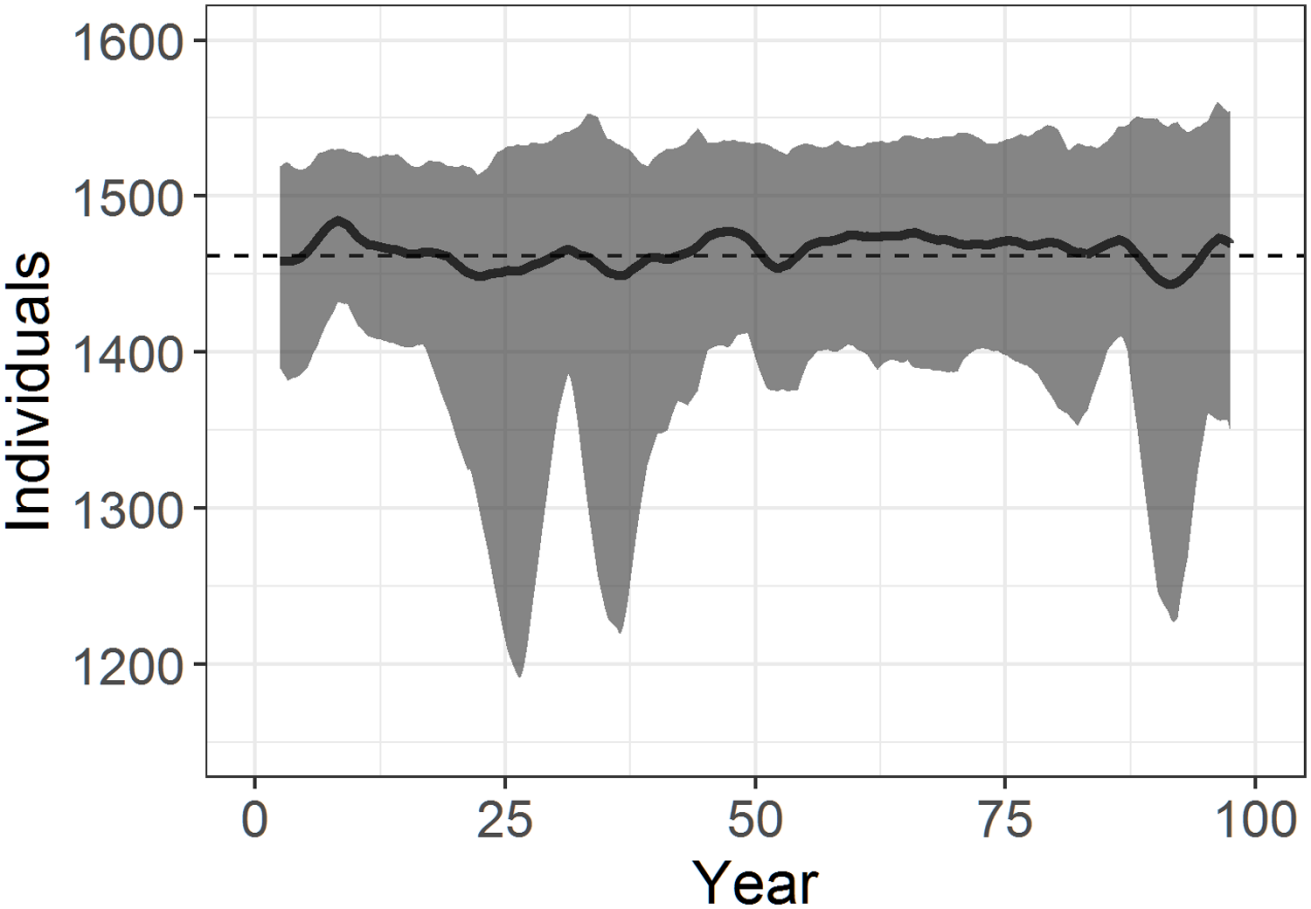
Model results of baseline population trajectory over 100 years. Baseline population showing 5-year running mean of 10 simulations over 100 years (excluding the run-in period), and overall population mean, at an initial food availability *f*_a_ = 0.935. The population remained stable at a mean of 1464±11 individuals (range 1191-1553). The grey enveloping the mean (black line) represents the minimum and maximum number of individuals in the population at each time step. The total mean population shows only independent individuals (*i.e*. juveniles and adults), as per Fig 4.

The baseline model (set with standard parameters as described in Tables 1, 2 and 3) produced populations that were stable over long periods of time (exceeding 2000 years). Fig 5 shows a mean stable population of independent (those not reliant on their mothers; *i.e*. juveniles and adults) seals, at 1464 individuals (±11, within a range of 1191-1553) over 100 years (from 10 simulations).

**Fig 6:**
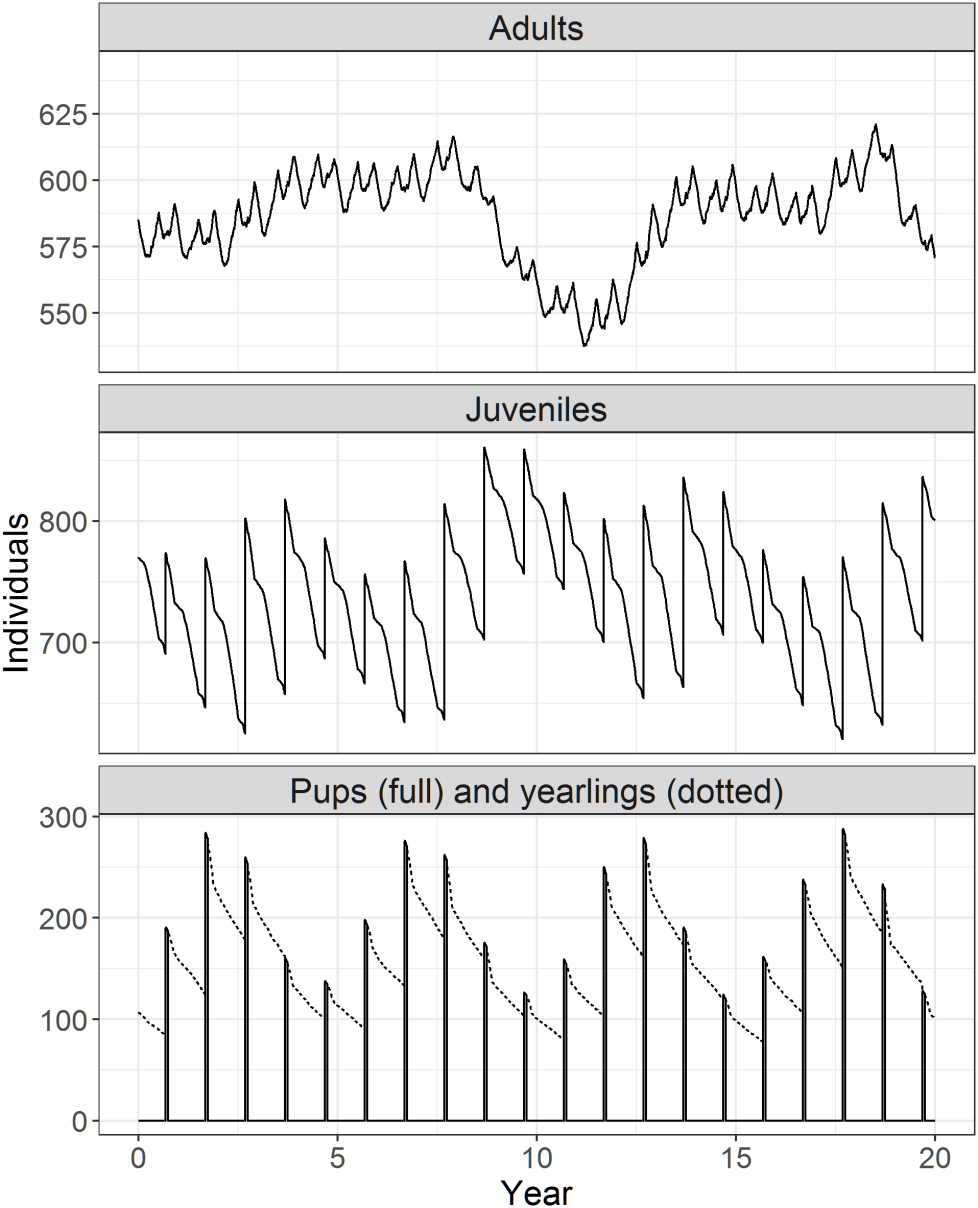
Population dynamics of baseline model. Population dynamics in the baseline model; mean of 10 runs, over 20 years. Individual stages as per transition thresholds, except juveniles do not include those under 1 year old—these are represented as yearlings in the third panel. The ‘Pups, and yearlings’ panel shows the survival at different stages (see also Table 4). Transition stages for adult: $U_{H}\geq U_{H}^{p}$, juvenile: $U_{H}^{x}\leq U_{H}<U_{H}^{p}$, pup: $U_{H}^{b}\leq U_{H}<U_{H}^{x}$. Note different scales on y-axis.

The population structure in the model is an emergent feature determined by the breeding success and survival of individuals. In the baseline model, these dynamics remained stable over time with the greatest proportion of the population being juveniles (Fig 6). Juveniles (excluding yearlings) and adults, annually, make up 49.83±0.71% and 39.51±0.74% of the population, respectively. Pup and yearling survival also remained stable over time at 97.55±0.36% and 65.76±2.17%, respectively (Table 4; columns 2 and 3 compare published observations with baseline model results for selected properties).

**Table 4:** Means from Monte Carlo simulations highlighting emergent life history and breeding behaviour of individuals, for baseline and sensitivity analyses. Means (±standard deviation) from Monte Carlo simulations (*n* = 10 over 100 years) for baseline model and sensitivity analyses. Grey cells indicate significant differences between means of baseline model and simulation runs (t-test; *p* <0.01). The changes applied to the model parameters are described in Table 3. * Indicates the distribution of selected model results are presented in Fig 7; ** indicates the distribution of selected model results are presented in Fig 8, and; *** indicates that the distribution of selected model result is presented in Fig 9. Results for lifespan of individuals exclude deaths of yearlings, and proportion of juveniles excludes counts of yearlings. ^a^ Females counted in 2015; modelled population is smaller to limit computational costs (see Discussion). ^b^ Indicates a significant difference in model results, although t-test failed as data has a reported variability of 0.00 (see also Results: Life history and breeding traits).

			Food availability		Weaning threshold		Puberty threshold	
	Observed data	Baseline model	Low	High	Low	High	Low	High
Population size **	2740 [39] ^a^	**1464±11**	425±2	1501±10	1470±12	1275±1	1474±33	1452±3
Juvenile proportion **	–	**49.83±0.71%**	60.1±0.20%	48±0.80%	55.6±0.50%	33±0.17%	31.1±0.75	72.6±0.78%
Adult proportion **	–	**39.51±0.74%**	29.7±0.19%	41.2±0.76%	33.3±0.57%	55.6±0.20%	58.1±0.97%	16.8±0.73%
Pup survival **	95% [71]	**97.55±0.36%**	96.2±1.37%	97.6±0.48%	97.6±0.38%	97.6±0.29%	97.6±0.35%	97.3±0.45%
Yearling survival *	54.2%-71.6% [51]	**65.76±2.17%**	43.2±2.92%	66.3±3.56%	73.5±1.04%	40±2.95%	59.8±3.12%	68.1±1.30%
Min reproductive age (yr)	3 [45]	**4.40±0.00**	5.40±0.00 ^b^	4.40±0.00	4.40±0.00	4.40±0.00	4.40±0.00	4.40±0.00
Generation time (yr) **	7.9-11.3 [68, 72]	**9.49±0.03**	11.48±0.00	9.52±0.05	9.59±0.07	9.46±0.00	9.48±0.02	9.47±0.00
Juvenile age (yr) *	–	**3.85±0.07**	4.58±0.01	3.57±0.10	4.13±0.07	2.57±0.00	2.36±0.02	5.5±0.08
Min adult age (yr) **	6 [45, 46]	**5.11±0.04**	8.88±0.04	5.01±0.02	5.18±0.03	4.66±0.02	3.97±0.02	6.49±0.05
Adult age (yr) *	–	**10.74±0.06**	13.80±0.04	10.47±0.08	10.50±0.15	10.23±0.03	9.43±0.19	12.10±0.08
Lifespan (yr) *	10-14 [31, 36]	**11.73±0.08**	12.88±0.07	11.49±0.08	11.09±0.14	12.20±0.07	11.67±0.27	11.76±0.06
Max lifespan (yr) ***	23 [47]	**28.80±0.99**	30.42±0.99	28.72±1.18	27.87±1.04	30.04±1.04	28.27±1.05	29.43±1.21
Fecundity *	0-0.5 [68]	**0.28±0.00**	0.35±0.00	0.28±0.00	0.28±0.00	0.39±0.00	0.31±0.01	0.26±0.00
Max pups per mum **	13 [69]	**8.90±1.10**	12.2±1.23	9.00±0.67	8.50±0.71	14.1±0.99	9.60±0.97	8.70±0.82
Juvenile size (cm) *	150-240 [73]	**168±0.16**	163±0.02	167±0.23	169±0.18	163±0.04	159±0.06	174±0.22
Adult size (cm) *	275 [74]	**188±0.31**	194±0.13	190±0.31	188±0.27	197±0.05	185±0.80	191±0.22
Max size (cm) *	280 [43, 44]	**193±0.59**	215±1.30	200±2.75	197±1.17	210±0.04	195±4.81	199±0.68
Effective food (age >360 d) **	–	**0.71±0.00**	0.88±0.00	0.71±0.00	0.71±0.00	0.82±0.00	0.68±0.02	0.74±0.00

The mean age at first successful breeding in the baseline model is at four years old (Table 4), with a generation time of 9.5±0.03 years (see section Life history and breeding traits in Results). First attempts at breeding are around the age of three; however the individuals generally have not reached the appropriate energy storage threshold to maintain these early pregnancies. The modelled individuals successfully reproduce up to 11 times within their lifetime, but often no more than nine. The mean fecundity (reproductive rate; *i.e*. number of female offspring per year [68]) of the population is 0.28 (range 0-1; Table 4), which is as expected considering the inclusion of the reproductive threshold *U*_cum_ to account for only female births in the model.

Individuals transition to adult stage at just over five years of age, with a mean lifespan of 11.73±0.08 years. The mean ages of juveniles and adults are 3.85±0.07, and 10.74±0.06 years, respectively. The mean maximum lifespan (from the absolute maximum ages reached by individuals in the model) sits at 28.80±0.99 years (see section Discussion: Lifespan and mortality). These estimates exclude the deaths of yearlings. The maximum size reached by individuals is 193±0.59 cm, with a mean of 168±0.16 cm and 188±0.31 cm for juveniles and adults, respectively (Table 4).

### Sensitivity analyses

**Fig 7:**
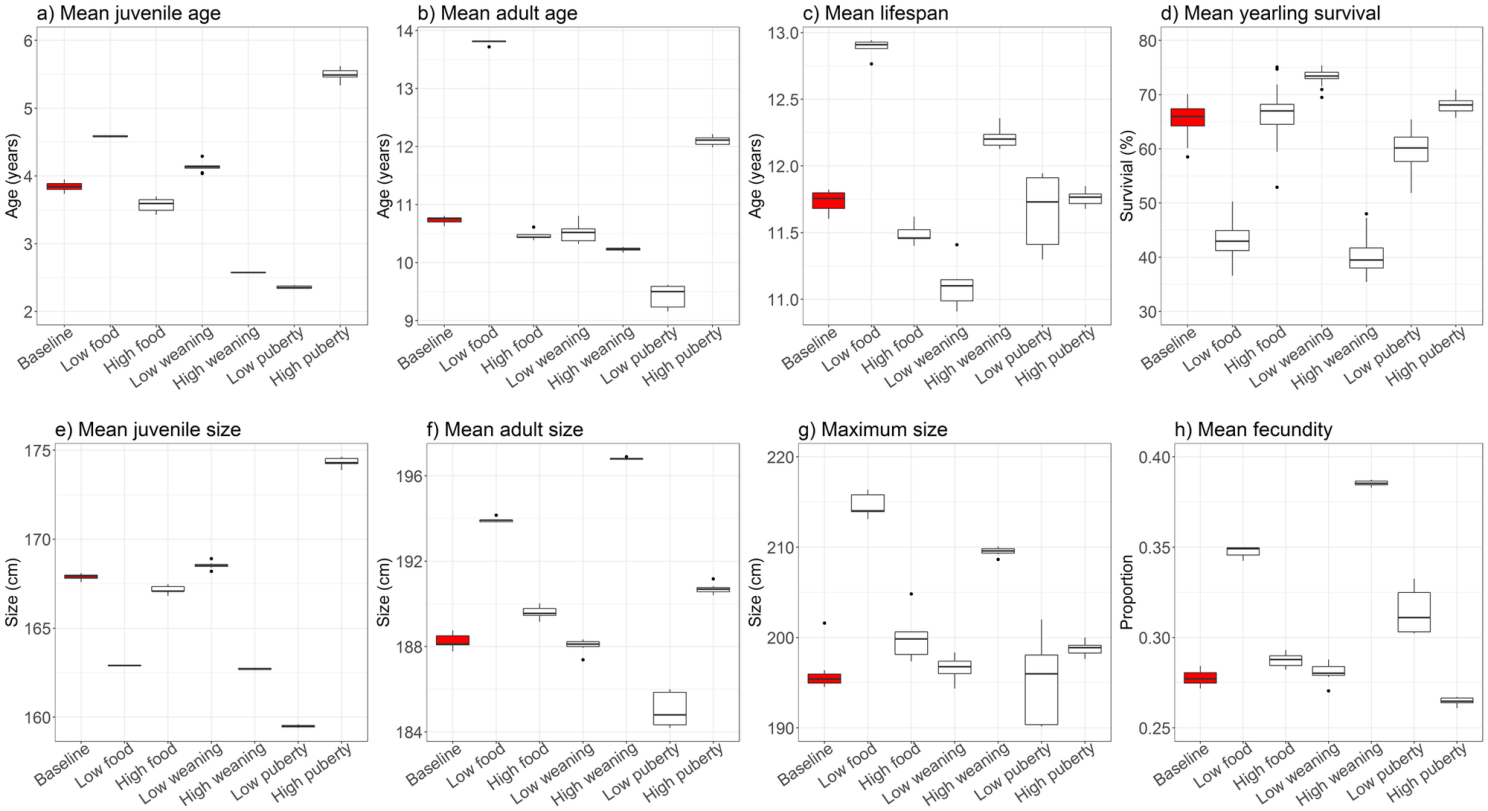
Comparison of results from Monte Carlo simulations showing emergent life history and breeding behaviour of individuals, for baseline and sensitivity analyses, part 1. Comparison of results from Monte Carlo simulations (means of *n* = 10, over 100 years), showing a) mean juvenile age, b) mean adult age, c) mean lifespan, d) mean yearling survival, e) mean juvenile size, f) mean adult size, g) maximum size, and h) mean fecundity of females in the baseline model and sensitivity runs. For each sub-plot the baseline result is highlighted in red. Results for means±standard deviation are found in Table 4.

**Fig 8:**
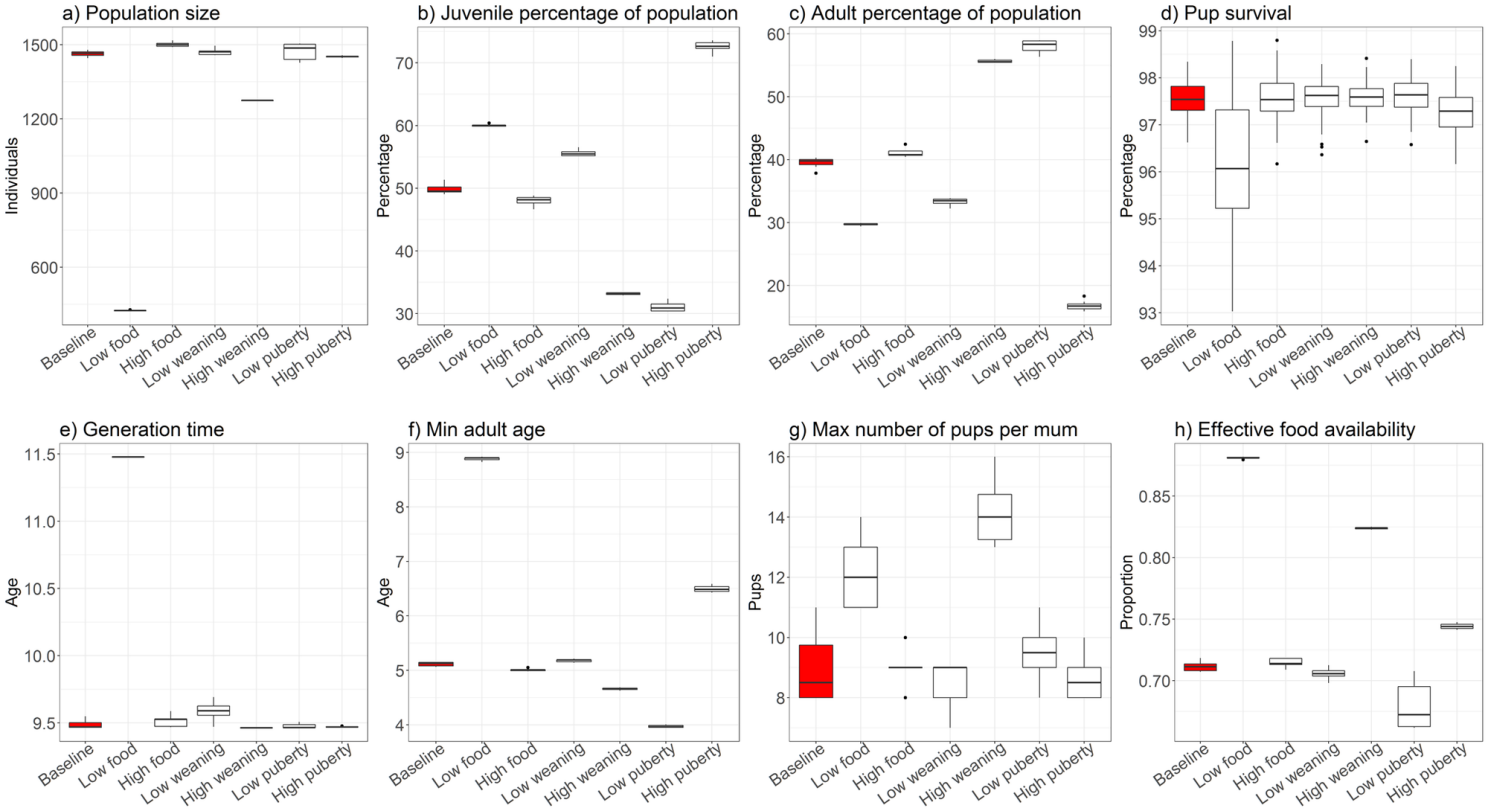
Comparison of results from Monte Carlo simulations showing emergent life history and breeding behaviour of individuals, for baseline and sensitivity analyses, part 2. Comparison of results from Monte Carlo simulations (means of *n* = 10, over 100 years), showing a) population size, b) juvenile percentage of population, c) adult percentage of population, d) pup survival, e) generation time, f) minimum adult (transition) age, g) maximum number of pups per mum, and h) effective food availability in the baseline model and sensitivity runs. For each sub-plot the baseline result is highlighted in red. Results for means±standard deviation are found in Table 4.

**Fig 9:**
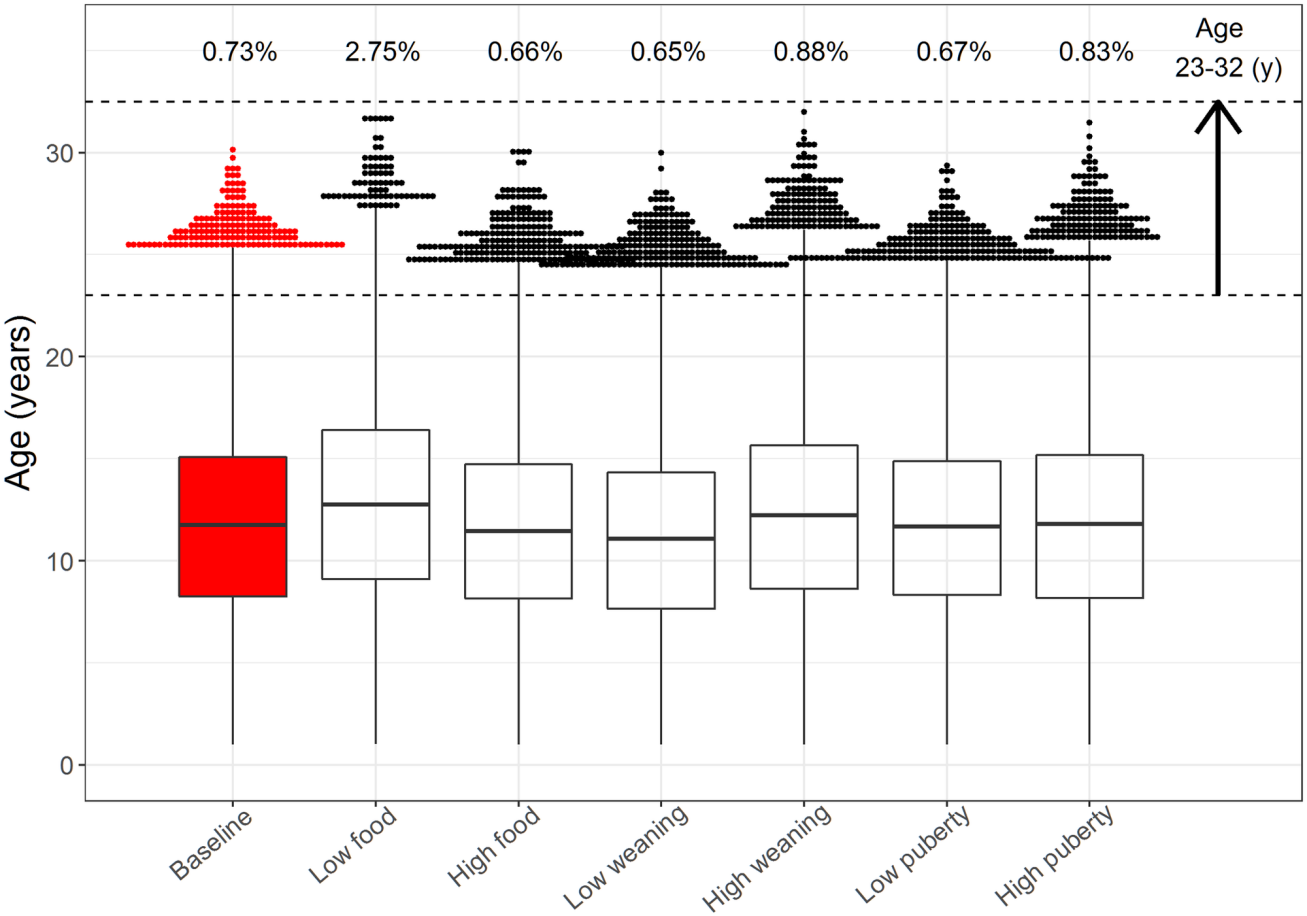
Population-level distribution of lifespan for baseline model and sensitivity runs. Population-level distribution of lifespan of individuals in the baseline and sensitivity model runs (100 years x 360 days x 10 runs). Outliers consist of ages 1.5 × the interquartile range, above the upper quartile. Individuals reaching ages >23 (the maximum recorded age in the field [47]) made up 0.73% in the baseline model (red; *n* = 107808); 2.75% and 0.66% with low and high initial food availability (*n* = 31488 and 110790, respectively); 0.65% and 0.88% with low and high weaning threshold (*n* = 10466 and 105817, respectively); and 0.67% and 0.83% with a low and high puberty threshold (*n* = 108412 and 107416, respectively). Deaths of yearlings have been removed from the analyses, and the baseline result is highlighted in red.

Results for sensitivity analyses for changes in initial food availability and transition thresholds at weaning and puberty are presented in Table 4. For ease of understating the scale and direction of change for the different parameters the results are visualised in Figs 7 and 8. Exceptions are made for the minimum reproductive age, which had little to no variation between results, and maximum lifespan which is presented in Fig 9.

#### Changes in initial food availability

Monte Carlo simulations indicated that mean population numbers were significantly different from the baseline model. Changes to the resource availability resulted in a mean population of 425±2 and 1501±10 individuals, respectively (Welch two sample t-test: *p* <0.01, *n* = 10; Table 4, Fig 8a) for a decrease (55%) and an increase (95%) of the initial food availability *f*_eff_.

At a lower initial food availability the proportion of adults and juveniles in the population changed significantly; >60% of the population are juveniles, and <30% adults (Fig 8b and 8c). Yearling survival dramatically reduced (to 43.2±2.92%; Fig 7d) as the fecundity increased (0.35±0.00; Fig 7h) and mothers gave birth to up to 14 pups in their reproductive lifespan (Fig 8g). The mean age of juveniles and adults, as well as their mean lifespan (Fig 7a–7c), increased and individuals took a year longer to become sexually reproductive (at 5.40±0.0 years old; Table 4). Juveniles transitioned to adults significantly later (at 8.88±0.04 years; Fig 8f) and individuals survived to almost 32 years of age (Fig 9). The mean juvenile size, however, reduced, which is in contrast to the mean adult and maximum sizes, which increased. The maximum size reached was 215±1.3 cm (Table 4, Fig 7e–7g).

At a higher initial food availability (*f*_a_ = 0.95) there was no change (Table 4) in the age at which individuals become sexually reproductive, nor was there a significant change in the proportion of juveniles (Fig 8b), the fecundity (Fig 7h), or yearling survival rates (Fig 7d). Although there were significant differences in the mean age and size of juveniles and adults, as well as for the age of transition to adult stage, and the proportion of adults in the population (Table 4), these differences were smaller than for a lower initial food availability (Figs 7a, 7b, 7e, 7f, 8c and 8f).

#### Changes in weaning threshold

A decrease in the weaning threshold $E_{H}^{x}$ significantly changed the population structure and dynamics (Table 4), with a greater proportion of the population being juveniles than adults (55.6±0.50% and 33.3±0.57%; Fig 8b and 8c), and a significant increase in the survival rate of yearlings (73.5±1.04%; Fig 7d), although there was no significant change in the mean population size (1470±12 individuals; Fig 8a). Monte Carlo simulations indicated significant increases in the mean ages and lifespan of individuals (Table 4), with the increase in mean juvenile age and decrease in mean lifespan being the most prominent (Fig 7a and 7c). There were no significant changes in the mean fecundity (Fig 7h) or maximum number of pups per mother (Fig 8g), and only juveniles showed a significant difference in the mean size (Fig 7e), compared to the baseline model.

A higher weaning threshold resulted in a significant reduction of the population size (1275±1; Fig 8a) as well as a significant reduction in the survival of yearlings (40±2.95%; Fig 7d), changing the dynamics to a population with 33±0.17% juveniles and >55% adults (Table 4; Fig 8b and 8c). Although there was no change in the minimum reproductive age, the mean adult and juvenile ages significantly reduced; juveniles transitioned to adult stage sooner (Fig 8f); and the lifespan and maximum ages increased (Fig 9). Fecundity also rose significantly (Fig 7h), and the mean number of pups produced by each mother increased to 14.1±0.99 (Table 4; Fig 8g). The mean juvenile size was significantly smaller than in the baseline model, however the mean adult and maximum sizes were significantly larger (Table 4, Fig 7e–7g).

#### Changes in puberty threshold

The mean population was not significantly different for either a decrease or an increase in the puberty threshold $E_{H}^{p}$, at 1474±33 and 1452±3 individuals, respectively (Table 4; Fig 8a). The population structure and dynamics, however, changed significantly. At a lower puberty threshold a significantly lower proportion of yearlings survived their first year (59.8±3.12%; Fig 7d), and the juvenile proportion of the population was 31.1±0.75%; with adults making up 58.1±0.97% of the population (Fig 8b and 8c). The age at transition to adult stage was more than a year lower than in the baseline model (Fig 8f); this is reflected in the mean juvenile and adult ages (Table 4, Fig 7a and 7b). The mean and maximum lifespan, however were not significantly different (Figs 7c and 9). There was no significant difference in the maximum size reached, however the mean juvenile size was lower, and the mean adult size higher, than those found in the baseline model (Table 4, Fig 7e–7g).

There were significant changes in the population dynamics following an increase in the puberty threshold, with an increase in the yearling survival rate (to 68.1±1.30%; Fig 7d) and a large increase in the juvenile proportion of the population (72.6±0.78%, compared to only 16.8±0.73% adults Fig 8b and 8c; Table 4). Additionally the age of transition (Fig 8f), as well as the mean juvenile and adult ages, increased significantly (Fig 7a and 7b). There was no significant change in the mean or maximum lifespan. The fecundity was significantly reduced (Fig 7h), although there was no significant difference in the maximum number of pups produced by each mother (Table 4; Fig 8g). The mean juvenile and adult sizes were significantly larger than the baseline results, although there was no significant difference in the maximum size reached (Table 4, Fig 7e–7g).

The maximum and absolute maximum lifespan from both the baseline model and the sensitivity analyses (Table 4) are higher than those observed in the field (23 years old [47]), <1% of the population in the baseline model reached a maximum age >23. In the simulation runs this ranges from 0.65-2.75% of the modelled population, overall (Fig 9).

## Discussion

The southern elephant seal DEB-IBM that we developed successfully replicated the general life-history and population behaviour of seals at Macquarie Island, while taking into account female births only. The model also illustrated how changes in food supply mediated through the size of pups at weaning affects population growth rates with positive rates associated with high weaning masses, and *vice versa*. This is important because for the first time we present information showing how environmental change is linked to individual animal performance; how performance affects vital rates (survival and fecundity), and; how changes in vital rates are manifested at the population level. We find that population growth rates are most sensitive to changes in survival rather than changes in fecundity, as might be expected for long-lived multiparous animals that place a higher premium on their own survival rather than that of their offspring [75].

The main goal of the project was to develop the first DEB-IBM for higher trophic-level species with complex life histories and to be able to simulate the energetic requirements of complex top predators in order to quantify how changes in the environment affect population growth rates and structure. We focused on female southern elephant seals as they have been part of extensive longitudinal studies on Macquarie Island, and census data on their life history and breeding traits are readily available. The ability to quantify prey consumption by predators is an essential component in ecosystem based management; as such a model that takes this as well as behavioural traits into account during the full year becomes a useful tool for management and conservation purposes. Our model shows that it is possible to have detailed energetics as well as behavioural traits included for higher trophic-level species in ecosystem models, through combining dynamic energy budget theory and individual-based modelling.

The sensitivity analyses were undertaken with changes to three model parameters. The changes to the weaning and puberty thresholds (the levels at which individuals are weaned, and physically become adults, respectively) were set at a 10% decrease and increase from the baseline (the standard parameter settings). Changes to the food availability parameter were made so that the lower limit (55% of available food) was set to represent an extreme scenario under which there was just over half the available food as is presented in the baseline model. The upper limit was set at 95% available food, as tests with an unlimited food supply were unfeasible as the model didn’t stabilise, predominantly due to computational limitations. These limitations were also the deciding factor regarding the set carrying capacity (or expected equilibrium) *K* of the model; at a stable population between 800-1600 individuals the model could be run overnight, and results can be compared to existing populations. For analyses of larger populations, we recommend a simple change to the model to include collectives, or super-individuals (*sensu* [76]), where one super-individual comprises multiple individuals, to limit computational costs.

### Life history and breeding traits

Life history traits (age at first reproduction, age at stage transition, maximum age, growth, and fecundity) are emergent behaviours in our model. The results of the baseline model are comparable with observations on Macquarie Island (see Table 4, and Figs 7 and 8), suggesting our model is successfully reproducing the behaviour of southern elephant seals. The behaviour, survival and breeding success of individuals ultimately affects the overall population structure and population trajectory.

#### Breeding

Females in the baseline model become adults around five to six years of age, and start reproducing around the age of four. This aligns with published data on ages at which individuals become sexually active and to which they undergo somatic growth [45, 46]. The generation time in the model is approximately 9.5 years; compared to 11.3 and 7.9 years previously estimated (respectively [68, 72]); where generation time is defined as the mean age of mothers at first birth [77]. Note that the observed minimum age at first successful reproduction has a reported variability of 0.00 (Table 4). This is as the analyses were undertaken on the means of the minimum age of each model run. Thus there were 10 means of the minimums, and considering that southern elephant seals have a short period during which they actually breed (at the same time every year) the mean minimum ages were identical.

The breeding behaviour of modelled individuals is mainly dependent on their stored reproductive buffer. If their accumulated reproductive buffer falls below the minimum breeding threshold, pregnant individuals will prioritise their own survival and abort their pregnancies; affecting their overall fecundity. Additional controls on reproduction are set through a chance of successful breeding that is dependent on age (*i.e*. a higher chance of reproductive success at four and five years [46, 60]; see Table 2). From conception through to weaning a pup’s energy intake is dependent on the mother’s energy stores [52, 60, 78]. This emphasises the importance of maternal foraging success [38] as up-regulation of energy intake during pregnancy is essential for mothers to be able to carry the offspring through to birth and weaning [18]. Consequently, a fitter mother will produce a bigger pup with a better chance of surviving to breeding age. In the field it is not unusual to observe seals that do not breed for a year, or at all [60]. Small females may abort before reaching full term, or may not get pregnant [48, 52, 60, 63].

Fecundity (or the reproductive rate) of southern elephant seals at Macquarie Island has previously been estimated to vary between 0 and 0.5 [68], indicating that not all seals breed every year. The emergent mean fecundity of each model simulation falls within that estimate (at 0.28), while taking into account female births only. The lowest resulting fecundity was seen in simulations of the model with a higher puberty threshold (at 0.26), and the highest fecundity was seen with a lower weaning threshold (at 0.39). This is a logical result as for simulations with a higher puberty threshold, individuals need to allocate more energy stores to their own growth and thus have less energy to allocate to breeding. The opposite is true for a lower weaning threshold, considering less energy needs to be allocated to personal growth while the pups are weaning. Consequently some of the energy gains may be allocated to the reproductive buffer sooner, resulting in an overall higher allocation of energy for breeding. This is reflected in the changes seen in the number of pups produced by these individuals in the model (10 and 16 pups, respectively) over their reproductive lifespan.

#### Ages at transition

Changes to the parameters for initial food availability and the transition threshold for weaning and puberty affected the emergent life history traits. A reduction of the available food affected the age at first reproduction (as described above); individuals started breeding later. This is not surprising considering a reduction in food means a reduction in energy intake, which therefore means it will take longer to reach energy related thresholds. Under scenarios with a lower initial food availability or a high puberty threshold, the age at which individuals transition from juvenile to adult stage (*i.e*. when they reach physical maturity) also occurs considerably later in life (at 8.88±0.04 and 6.49±0.05 years, respectively). This increase in the transition age is reflected in the higher mean juvenile and adult ages (Fig 7a and 7b).

Simulations with a higher initial food availability, an increase in the weaning threshold, and a decrease in the puberty threshold had no effect on the age at first breeding, but did have significant effects on the ages at which individuals became adults. Particularly simulations with a high weaning or low puberty threshold reduced the age at transition (to 4.66±0.02 and 3.97±0.02 years, respectively). This is explained by the different allocation of energy storage for physical maturation *U*_*E*_ and the reproductive buffer *U*_*R*_ where the energy allocated to reproduction is not affected by changes in the transition thresholds, thus the age at first breeding does not change. With a reduced puberty threshold, the individuals became physically mature before they became sexually reproductive.

### Pup and yearling survival

The mean annual pup survival in the baseline model (taking into account the combined energetic and non-energetic mortality for pups and yearlings) is 97.55±0.63% for pups (while with their mother) and 65.76±2.17% for yearlings after weaning (Table 4, Fig 7d). No records are published on the survival rates of pups during the lactation period, however, it is estimated that approximately 5% die during this period [71], due to being squashed by either their mother, or other adults on the beach. This is not explicitly included in the model, but emerges from the non-energetic mortality factor applied to pups and yearlings.

The yearling survival rate lies within the size dependent range observed for yearlings at Macquarie Island (54.2% to 71.6% [51]). The survival rate of yearlings varied significantly between the sensitivity runs of the model (ranging from 40±2.95% to 73.5±1.04%, respectively, for a higher and lower weaning threshold). These extremes are reasonable considering the energetic mortality threshold of yearlings is closely related to their weaning threshold (see section Sub-models in Materials and methods, above) and while this is changed in the model, there is no change implemented to the energy intake in the same period. Consequently yearlings in the model where there is an increase in the weaning threshold may not be able to sustain these high energy levels, and die. Those individuals who do survive through to sexual maturity, are more successful at breeding (indicated by the highest fecundity rate; see above) reflecting the observed survival differences between small and large pups on Macquarie Island (*e.g*. [51]).

#### Lifespan and mortality

There were no significant differences in the maximum lifespan of individuals in the sensitivity runs, compared to the baseline (although a lifespan of close to 2 years longer for a lower initial food availability, and an increase in the weaning threshold gave a *p*-value of 0.028 and 0.014, respectively). The maximum lifespan of individuals in the model is higher than the maximum ages observed on Macquarie Island, however, the percentage of individuals with higher ages was low (range of 0.65-2.75% between simulations; Fig 9). As initial tests of the model showed that these few animals in the older age classes contribute very little to the overall population parameters, we made the decision not to add an absolute maximum age to the model at which individuals were forced to die, but for the maximum age to remain an emergent feature.

Changes in the puberty threshold did not change the mean lifespan of individuals, whereas both changes in the initial food availability and the weaning threshold did. The mean lifespan of individuals increased for a higher weaning threshold, as well as with lower initial food availability, as did the maximum and absolute maximum lifespan in these scenarios. This is not unreasonable when looking at research on effects of calorie restriction on lifespan of a range of species, although opinions vary [79, 80]. This calorific constraint at a lower initial food availability would be an oscillating occurrence, parallel to the variations in population size, and consequent effective food availability (see section Population size and dynamics, below).

### Individual growth

The maximum size that individuals reached in the baseline model, as well as for each of the sensitivity runs, is lower than the field observations at Macquarie Island (195-215 cm; Table 4, and 280 cm; Table 1, respectively), although the modelled mean juvenile size sits within the predicted range (150-240 cm [73]). The lower size is likely to be due to changes made to the transition threshold for puberty in the model development stage to account for a more realistic age at which individuals reach physical maturity and become adults (see section Thresholds for puberty, breeding and death in Materials and methods). In simulations with a lower puberty threshold, the individuals had a lower mean and maximum size than the baseline (Fig 7e–7g), thus following the same trends as the changes observed in the baseline, compared with field observations (*i.e*. lower sizes for a lower puberty threshold). This is particularly clear in the differences for mean juvenile size (Fig 7e), and can be related back to the younger age at which these individuals become adults (Figs 7b and 8f), and *vice versa* for an increase to the puberty threshold.

An increase in the food availability resulted in larger adults compared to the baseline model, and a decrease in the available food also resulted in significantly larger adults (Fig 7f and 7g), although juveniles in both simulations remained smaller than in the baseline model (Fig 7e). This may be explained by the changes in the effective food availability, which increased at smaller populations—consequently producing individuals who (while not under periods of calorific constraint; see section Population size and dynamics, below) would grow faster and larger than their counterparts.

### Population size and dynamics

The number of individuals at which the population stabilises is partially dependent on the competition term Δ*P* (eq 3); this implements self-limitation to the population and maintains a stable, density regulated population, as is observed on Macquarie Island [81]. The competition takes into account the carrying capacity (or expected equilibrium) *K*, the current population *P*, and the initial food availability *f*_a_. A stable population is maintained at an effective food availability *f*_eff_ somewhere between 0.75-0.9. There is variability in the modelled population caused by changes in the effective food availability, as individuals enter and leave the population. This fluctuation has also been observed in the field, and is thought to be related to the effects of climate variability at foraging grounds and the consequent changes in food availability observed three years prior [82].

## Conclusion and next steps

The DEB-IBM we developed for southern elephant seals produced a biologically realistic, stable population, where individuals reproduce at the expected age, finish somatic growth (reach physical maturity) after reaching sexual maturity and reach the observed life expectancy (based on expectations from the Macquarie Island population). The model can be used as a stand-alone, single species model for projecting effects of intrinsic and extrinsic changes on individuals and the population through analyses of behaviour and energy use. The model is developed in such a way that, with relative ease, it could be implemented for other seal species, or a range of other marine mammals or birds.

Our model is not spatially resolved, and as such we do not have a prey-field. Instead, we have a value for initial food availability *f*_a_ (currently set at 0.935). The exclusion of males makes little difference in this case, as the initial food availability can be simply adjusted to produce a stable population with either just females or males and females. To make the model more realistic, the currently used relative (analytical) food availability could be modified so that more realistic prey fields are included in the model (see *e.g*. [16, 17]). If we develop a spatially explicit version of this model then the presence of males becomes more important as their different foraging patterns may impact food availability differently. Including a more realistic prey field, and making the model spatially explicit, would also include adding prey dependent energy densities, improving the accuracy of the predator’s energy intake and use at different times and locations. A detailed sensitivity analysis is recommended for development of a spatially explicit DEB-IBM for southern elephant seals, as energetic intake and requirements may change (particularly with implementation of actual foraging behaviour). This may alter the results to some extent, based on the sensitivity of this model to changes in resource availability and transition thresholds.

Future development of this model could include explicitly modelling male births in the model and, when the model is spatially explicit, modelling the southern elephant seal population in its entirety. This would involve lowering the energetic cost of birth to ensure mothers produce a 1:1 ratio of female and male pups, and increase fecundity closer to 0.5. However, including explicit representation of males will make the entire model more complex considering they have different foraging patterns, different energetic costs associated with different growth, age of physical and sexual maturity, as well as different mortality rates (as explained in the Introduction). Consequently the simplest solution to having male births included in the model, without increasing the complexity too much, would be to remove males after weaning. This would ensure that the mother’s energy expenditure on births will be more accurate than in the current model, however complexity in the model, regarding different life histories of males and females, will be limited. The only time we should consider it is if we have a spatially explicit prey-field.

Further modifications to the model could allow DEB-IBMs to be coupled with end-to-end ecosystem models to improve the representation of top predators through inclusion of detailed behavioural traits as well as energetic requirements. As such it could be used to infer management decisions for relevant fisheries, or for ecosystem management. As it stands now, we show that the complex life histories of southern elephant seals can be represented using DEB-IBMs. This model can project population dynamics which can be used to obtain a better understanding of potential drivers behind changes in populations.

## Supporting information

S1 Table Life table of southern elephant seals at Macquarie Island. Comparison of survival rates and relative numbers of males and female southern elephant seals on Macquarie Island derived from capture-mark-recapture studies. Hot-iron brands were used to individually and permanently mark seals. A combination, alpha-numeric brand was applied in different orientations on the seals to uniquely identify each individual [83, 84] over long periods [85] without any deleterious life-history affects [84, 86, 87, 88]. Maximum observed age of males is 15 years old, and females is 23 years old.
(PDF)
