# Supplementary material for: Modelling southern elephant seals Mirounga leonina using an individual-based model coupled with a dynamic energy budget
Source: PLoS One. 2018 Mar 29;13(3):e0194950. doi: 10.1371/journal.pone.0194950 (PMC5875804; doi:10.1371/journal.pone.0194950)
Supplement: S1 Table — Comparison of survival rates and relative numbers of males and female southern elephant seals on Macquarie Island derived from capture-mark-recapture studies. Hot-iron brands were used to individually and permanently mark seals. A combination, alpha-numeric brand was applied in different orientations on the seals to uniquely identify each individual [83, 84] over long periods [85] without any deleterious life-history affects [84, 86, 87, 88]. Maximum observed age of males is 15 years old, and females is 23 years old. (PDF) [file pone.0194950.s001.pdf]

Supporting Information to Goedegebuure *et al.* (2018)

DOI 10.1371/journal.pone.0194950

S1 Table : Life table of southern elephant seals at Macquarie Island. Comparison of survival rates and relative numbers of males and female southern elephant seals on Macquarie Island derived from capture-mark-recapture studies. Hot-iron brands were used to individually and permanently mark seals. A combination, alpha-numeric brand was applied in different orientations on the seals to uniquely identify each individual [1, 2] over long periods [3] without any deleterious life-history affects [2, 4, 5, 6]. Maximum observed age of males is 15 years old, and females is 23 years old.

Age	Male survival	Number of males	Female survival	Number of females
0	1	2000	1	2000
1	0.671	1342	0.696	1392
2	0.534	1068	0.568	1136
3	0.438	876	0.463	926
4	0.361	722	0.387	774
5	0.275	550	0.335	670
6	0.201	402	0.267	534
7	0.151	302	0.208	416
8	0.118	236	0.166	332
9	0.088	176	0.138	276
10	0.058	116	0.114	228
11	0.035	70	0.087	174
12	0.02	40	0.064	128
13	0.008	16	0.048	96
14	0.006	12	0.037	74
15	0.002	4	0.029	58
16			0.022	44
17			0.018	36
18			0.014	28
19			0.011	22
20			0.008	16
21			0.006	12
22			0.005	10
23			0.004	8

References

1. Chittleborough RG, Ealey EHM. Seal Marking at Heard Island, 1949, in: Law, P.G. (Ed.), ANARE Interim Report 1. Antarctic Division, Department of External Affairs., Melbourne, 1951; 1–30

2. McMahon CR, Burton HR, van den Hoff J, Woods R, Bradshaw CJA. Assessing hot-iron and cryo-branding for permanently marking southern elephant seals. Journal of Wildlife Management. 2006; 70(5): 1484–1489 doi:10.2193/0022-541X(2006)70[1484:AHACFP]2.0.CO;2

3. Hindell MA, Little GJ. Longevity, fertility and philopatry of two female southern elephant seals (Mirounga leonina) at Macquarie Island. Marine Mammal Science. 1988; 4(2): 168–171 doi:10.1111/j.1748-7692.1988.tb00197.x

4. McMahon CR, Bradshaw CJA, Hays GC. Branding can be justified in vital conservation research. Nature 2006; 439: 392–392. doi:10.1038/439392c

5. McMahon CR, Bradshaw CJA, Hays GC. Applying the heat to research techniques for species conservation. Conservation Biology 2007; 21(1): 271–273. doi:10.1111/j.1523-1739.2006.00566.x

6. van den Hoff J, Sumner MD, Field IC, Bradshaw CJA, Burton HR, McMahon CR. Temporal changes in the quality of hot-iron brands on elephant seal (Mirounga leonina) pups. Wildlife Research 2004; 31(6): 619–629. doi:10.1071/WR03101
